# Environmental and political implications of underestimated cropland burning in Ukraine

**DOI:** 10.1088/1748-9326/abfc04

**Published:** 2021-05-21

**Authors:** Joanne V Hall, Sergiy V Zibtsev, Louis Giglio, Sergii Skakun, Viktor Myroniuk, Oleksandr Zhuravel, Johann Georg Goldammer, Nataliia Kussul

**Affiliations:** 1 Department of Geographical Sciences, University of Maryland, 2181 LeFrak Hall, College Park, MD 20742, United States of America; 2 Institute of Forestry and Landscape-Park Management, National University of Life and Environmental Sciences of Ukraine, Regional Eastern Europe Fire Monitoring Center (REEFMC), 15 Heroiv Oborony Street, Kyiv 03041, Ukraine; 3 Food and Agriculture Organization of the United Nations, Regional Office for Europe and Central Asia, 26 Esplanadna Street, Kyiv 02000, Ukraine; 4 Global Fire Monitoring Center (GFMC), Max Planck Institute for Chemistry and Freiburg University, Georges-Koehler-Allee 75, D-79110 Freiburg, Germany; 5 Department of Space Information Technologies and Systems, Space Research Institute NAS Ukraine & SSA Ukraine, Kyiv 03680, Ukraine; 6 Equally contributing second authors.

**Keywords:** crop residue burning, active fires, VIIRS, remote sensing, Ukraine, policy

## Abstract

Open burning is illegal in Ukraine, yet Ukraine has, on average, 300 times more fire activity per year (2001–2019) than most European countries. In 2016 and 2017, 47% of Ukraine was identified as cultivated area, with a total of 70% of land area dedicated to agricultural use. Over 57% of all active fires in Ukraine detected using space-borne Visible Infrared Imaging Radiometer Suite (VIIRS) during 2016 and 2017 were associated with pre-planting field clearing and post-harvest crop residue removal, meaning that the majority of these fires are preventable. Due to the small size and transient nature of cropland burns, satellite-based burned area (BA) estimates are often underestimated. Moreover, traditional spectral-based BA algorithms are not suitable for distinguishing burned from plowed fields, especially in the black soil regions of Ukraine. Therefore, we developed a method to estimate agricultural BA by calibrating VIIRS active fire data with exhaustively mapped cropland reference areas (42 958 fields). Our study found that cropland BA was significantly underestimated (by 30%–63%) in the widely used Moderate Resolution Imaging Spectroradiometer-based MCD64A1 BA product, and by 95%–99.9% in Ukraine’s National Greenhouse Gas Inventory. Although crop residue burns are smaller and emit far less emissions than larger wildfires, reliable monitoring of crop residue burning has a number of important benefits, including (a) improving regional air quality models and the subsequent understanding of human health impacts due to the proximity of crop residue burns to urban locations, (b) ensuring an accurate representation of predominantly smaller fires in regional emission inventories, and (c) increasing awareness of often illegal managed open burning to provide improved decision-making support for policy and resource managers.

## 1. Introduction

**Figure 1.**
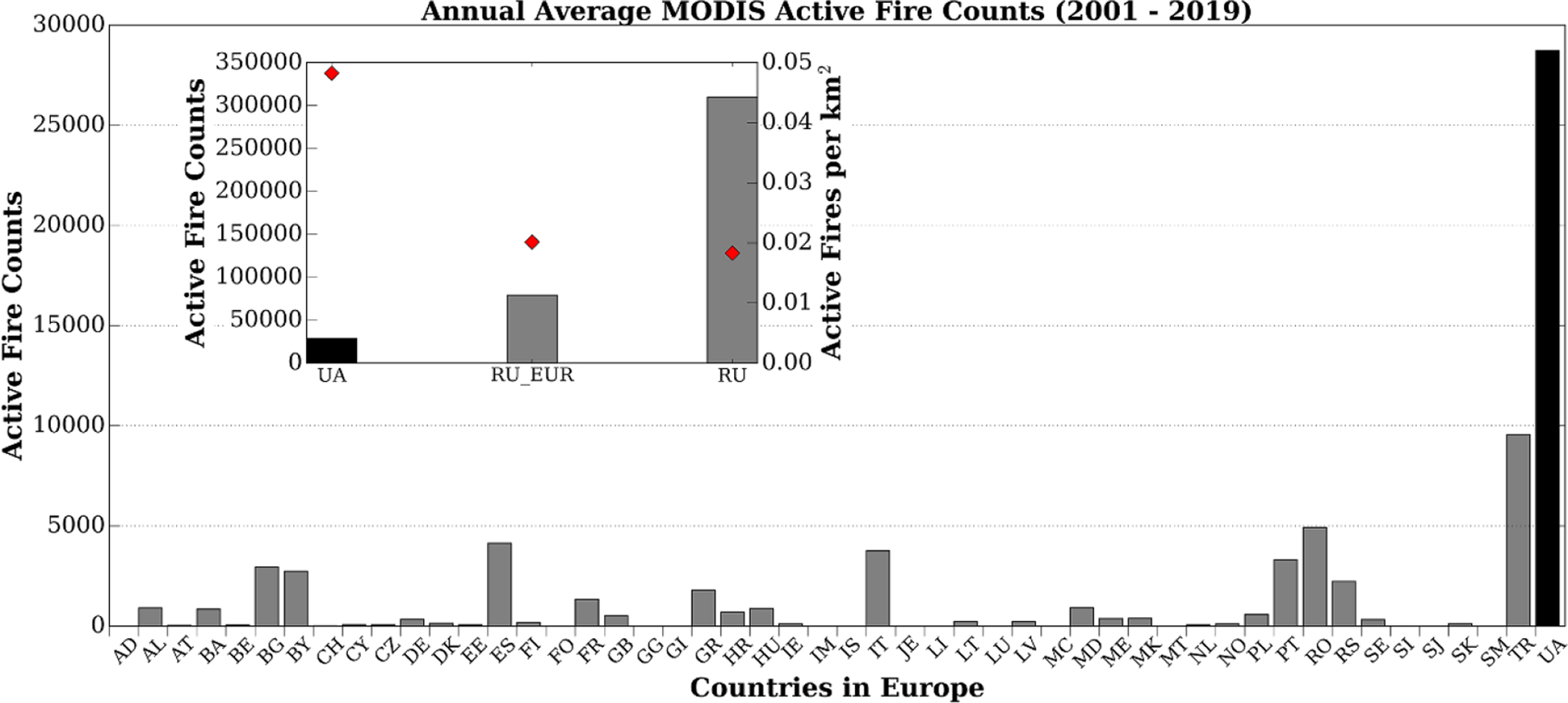
Annual average (2001–2019) MODIS active fire pixel counts for all European countries. Ukraine (black bar) has the highest annual active fire count on average compared to any other European country. The inset map compares the number of MODIS active fire pixels within Ukraine (UA), European Russia (RU_EUR), and all of Russia (RU). The red diamonds show the number of average annual MODIS active fires per km^2^. Data source [5].

During the last two decades, open burning of agricultural residues has been banned in all European Union (EU) countries under EU regulation 1306/2013 [1]. This widespread ban is a primary outcome of the implementation of EU renewable energy policies aimed at increasing the use of biofuel to achieve 20% of the total energy production from non-fossil fuel sources by 2020, coupled with various climate change policies aimed at reducing carbon emissions in the EU-member countries [e.g. 2–4]. The vast majority of these countries strictly follow the EU no open burning policy, including three Baltic countries—Estonia (EE), Latvia (LV), and Lithuania (LT)—that drastically reduced open burning after becoming full members of the EU (figure 1). For example, Lithuania’s burned area (BA) mapped using the Moderate Resolution Imaging Spectroradiometer (MODIS) decreased from over 60 km^2^ in 2002 and 2003 to a median value (2004–2019) of 6 km^2^ since joining the EU in 2004 (https://gwis.jrc.ec.europa.eu/apps/country.profile/charts).

While there is a clear increase in the amount of open burning in some non-EU countries within the Balkans and Eastern Europe, this increase is eclipsed when compared to the large scale open burning in Ukraine (UA) and European Russia (RU_EUR). For example, Turkey (TR) has seen an increasing trend in annual MODIS BA and active fire counts since 2002 with a maximum of *~*13 000 km^2^ (*~*5600 fire counts) of BA in 2009 (1.7% of the country area) compared to a maximum of *~*45 000 km^2^ (*~*53 000 fire counts) in Ukraine in 2008 (7.5% of the country area). In addition, the higher number of average annual MODIS active fires per km^2^ in Ukraine compared to the whole of Russia (RU)—where there are a substantial number of wildfires spread over large areas—further highlights the scale of this problem (figure 1). Therefore, it is imperative to ensure the extent of open burning within Ukraine is accurately quantified to help support the national strategies focused on reducing open burning to EU-levels.

**Figure 2.**
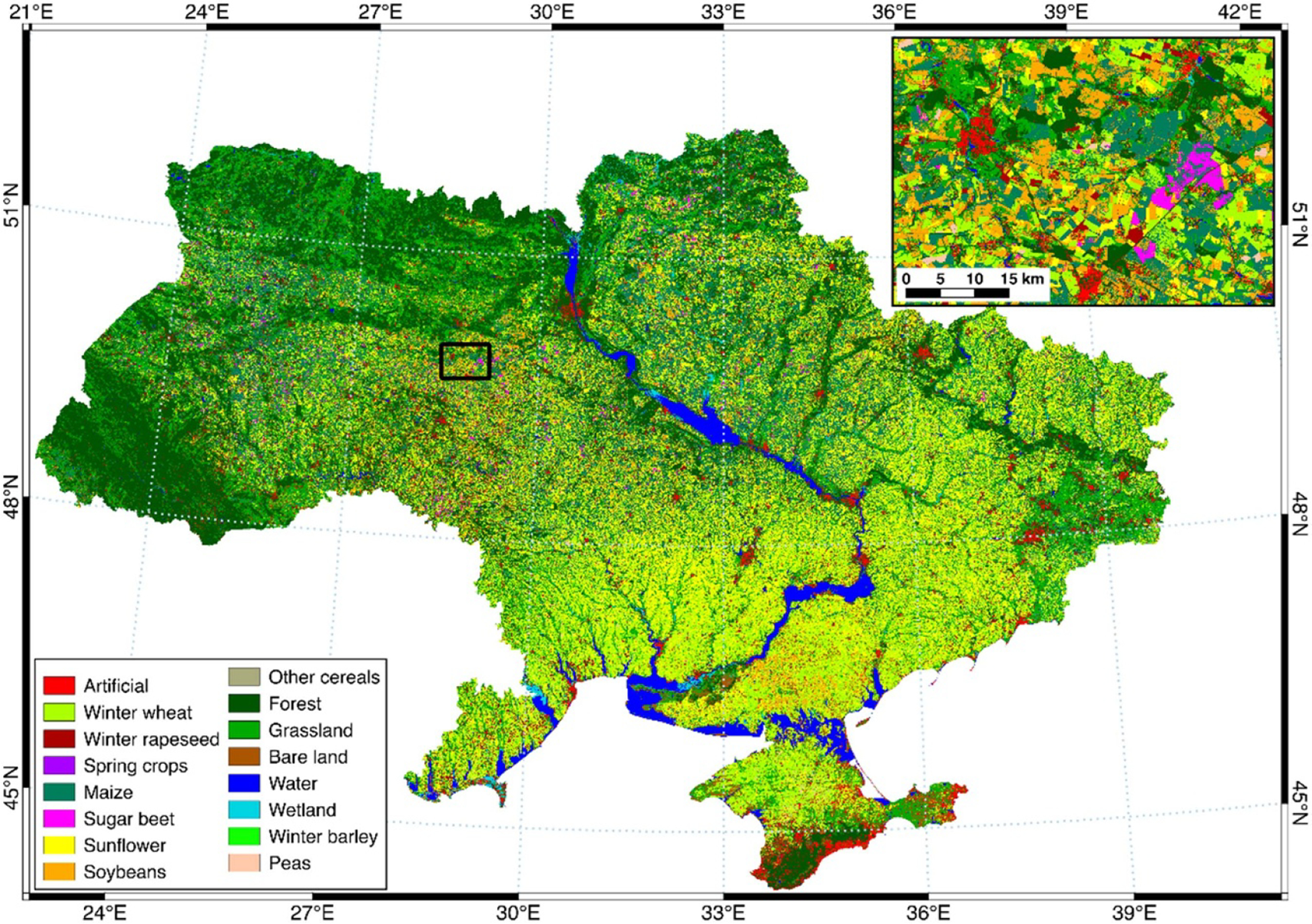
2017 Ukraine land cover/land use classification map (10 m resolution). Data source [12]: (supplementary text S1.0 and table S1).

Ukraine is one of the major global agricultural producers and exporters with approximately 70% of the land area dedicated to agricultural use (cropland, pastures, meadows etc). In 2016 and 2017, 47% of Ukraine was identified as cropped/sown area—i.e. land used for arable production (figure 2). A large proportion of open burning in Ukraine is associated with post-harvest crop residue burning and field clearing before planting. At present, the burning of crop residue in Ukraine is prohibited under the Code of Administrative Offenses (Article 77-1), Criminal Code (Article 241 and 245), and the Law of Ukraine on Air Protection (Article 20) [6–8]. Before the April 2020 legislative changes, violators faced fines of between 5100–8500 Hryvnia (*~*$200–$340 US) and up to 5 years in prison; the fines have since increased to *~*$230–$5880 US. In addition, if a burn becomes uncontrollable and causes either fatalities or infrastructure damage, violators can face up to 10 years in prison. Despite these penalties, Ukraine has on average *~*300 times more active fire pixel counts per year than most other European countries and has the highest active fire count per unit area even when compared to Russia (RU) (figure 1; based on MODIS MCD14ML Collection 6 Active Fire product [5]). This widespread practice has continued despite attempts by the government to address open burning and subsequent emissions in its agriculture, environmental, and population health protection policies [9, 10]. The absence of accurate, scientifically-based national assessments of burning has led to numerous country-level and international reports underestimating the severity of this problem (e.g. United Nations Framework Convention on Climate Change (UNFCCC) Ukraine’s National Greenhouse Gas Inventory [11]; see section 3 for a detailed description). Furthermore, several unresolved problems have further exacerbated this issue, including the opening of the land market, and a lack of policy support (e.g. subsidies for farm equipment that dispose of crop residue without the need to burn them) for agriculture and rural development.

In 2001, a ban on buying and selling certain types of agricultural land was implemented in Ukraine, with the result that the majority of land users now rent their lands and therefore have no investment in the long-term quality of the soil. With the recent March 2020 decision to open the land market in Ukraine in 2021 [13], it is imperative to understand the extent of agricultural burning and land management from a local and regional standpoint to help ensure targeted resource management in the regions with the greatest open burning. Excessive crop residue burning has a wide range of implications within both the immediate vicinity of the fire as well as further afield. For instance, within areas with excessive crop residue burning there are numerous examples in the scientific literature highlighting the impacts of open burning on local air quality and human health issues [e.g. 14–17], decreased soil health and crop yields [e.g. 18–20], and damages from out of control burns [e.g. 21, 22]. In addition, studies have also shown that short-lived climate pollutants emitted from cropland burning as far south as the southern extent of European Russia (~40*°* N) can be transported and deposited in the Arctic during seasons when land and sea ice melting can be affected by deposition of absorbing particles on the ice surface [23–25]. Therefore, minimizing open burning in Ukraine will have numerous positive environmental and human-health effects beyond the cropland regions.

Satellite-based cropland BA mapping is notoriously difficult due to the small fire sizes and rapid changes within each field during the transition from harvesting to planting [26–31]. In addition, each country has its own unique challenges to mapping cropland burning. Typically, field size (subsistence versus commercial farming), burn type (full field burn versus pile burning), geographical location relative to the orbital overpasses (high latitudes have increased orbital overlap from polarorbiting sensors), cloud cover persistence, and crop type (e.g. rice versus sugarcane fires) are just some of the challenges associated with mapping cropland burns. Ukraine is unique in that it offers a number of advantages over other countries with extensive and underreported crop residue burning (e.g. India, Thailand), including relatively large field sizes (average mapped field size = 0.4 km^2^; supplementary table S2 (available online at stacks.iop.org/ERL/16/064019/mmedia)), multiple orbital overpasses, and comparatively intense burns due to high yield. However, the black Chernozem soil causes additional complications when using traditional spectral reflectance-based BA mapping methodologies [26]. Unfortunately, since open burning is illegal in Ukraine, traditional ground-based surveys and even state-derived statistics are highly susceptible to under-reporting. Consequently, despite the complications noted above, remote sensing techniques offer an appealing route toward accurately quantifying the level of crop residue burning within Ukraine.

## 2. Methods

Here we describe a new technique to estimate cropland BA in Ukraine by calibrating 375 m Visible Infrared Imaging Radiometer Suite (VIIRS) active fire observations (VNP14IMGML [32]) with exhaustively mapped BA estimates within predefined reference areas. Highly detailed 10 m land cover/crop type maps [12] (available in 2016 and 2017) were used to determine both the extent of Ukraine’s cropland area and the crop types, therefore, our study was limited to 2016 and 2017.

### 2.1. Field mapping

**Figure 3.**
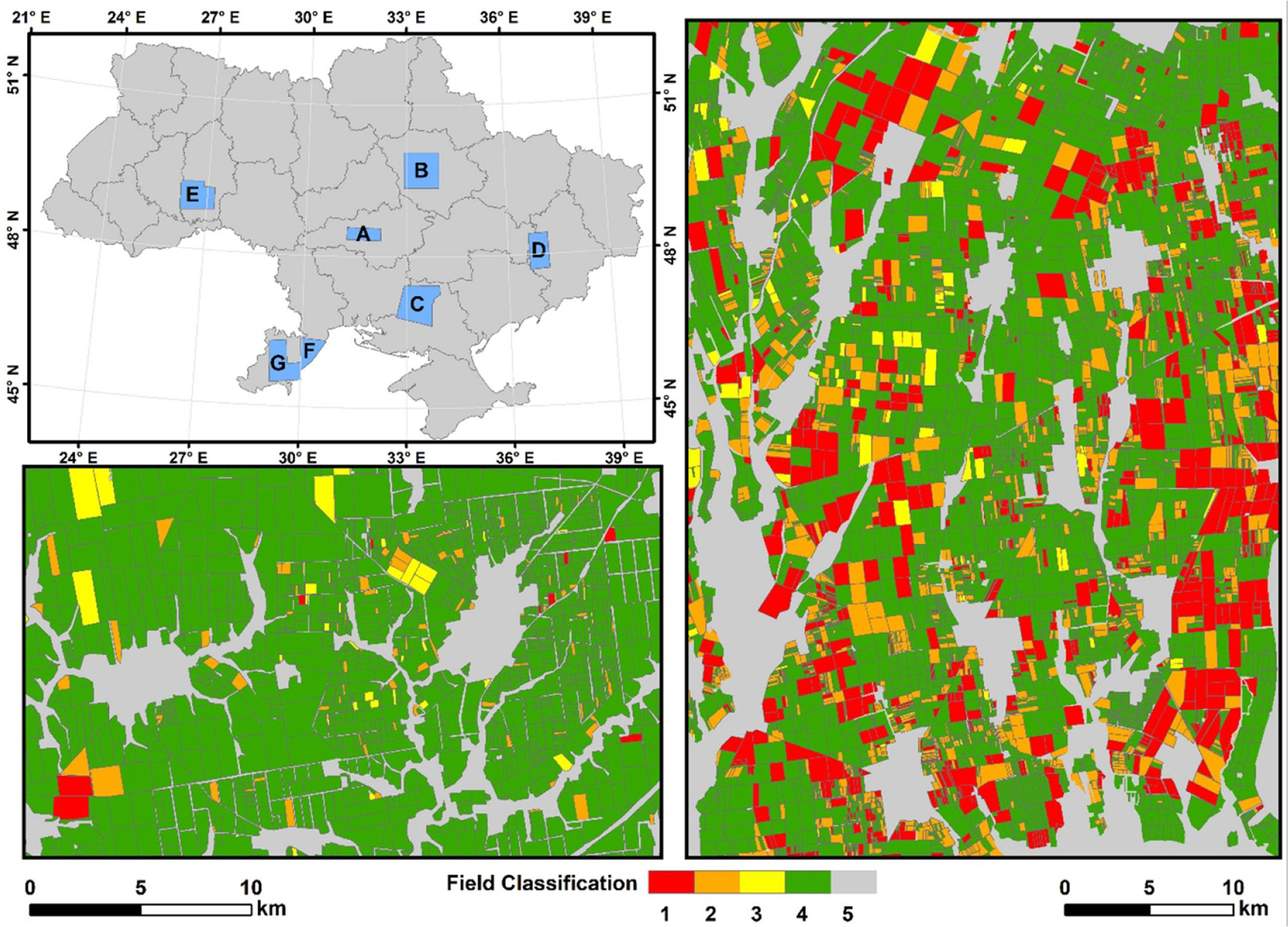
Seven mapped reference areas within Ukraine croplands (A–G; top left panel). Two enlarged panels illustrate the detail within a springtime, predominantly maize, area (location A; bottom left panel) and a summertime, predominantly winter wheat, area (location G; right panel). All polygons were attributed with the following field classification: 1 = active flame or BA with corresponding VIIRS active fire point; 2 = definite BA but with no flame or active fire point; 3 = ambiguous (a distinct darkening occurred on the field, but analyst is unsure if the field was burned then plowed or only plowed); 4 = definitely unburned; 5 = non-cropland or fields are too small that land cover conditions were difficult to determine on very high resolution (3 m) imagery. supplementary text S3.0, figures S2–S8, and table S2 provides a full description of each mapped reference area.

A team of analysts manually digitized cropland field boundaries and determined burned/unburned classifications for fields within seven reference areas that covered almost 5% of Ukraine’s land area (figure 3 and table 1). All analysts were trained by the project lead and quality checks were performed throughout the mapping process. Fields were classified using a combination of 20 m Sentinel-2 Multi-Spectral Instrument, 30 m Landsat-8 Operational Land Imager, and 3 m Planet imagery (www.planet.com), in conjunction with filtered VIIRS active fire point data (VNP14IMGML [32], supplementary text S2.0 provides detailed information on the active fire filtering). All polygons were attributed with the following classifications: 1 = active flame or BA with corresponding VIIRS active fire point; 2 = definite BA but with no flame or active fire point; 3 = ambiguous (a distinct darkening occurred on the field, but analyst is unsure if the field was burned then plowed or only plowed); 4 = definitely unburned; 5 = non-cropland or fields are too small that land cover conditions were difficult to determine on very high resolution (3 m) imagery. Each polygon was also attributed the following information: BA percentage (visually determined by the analyst for classes 1, 2, and 3), date of burn (class 1; class 2 if possible), and a ‘no area’ flag (a polygon was flagged if the analyst was unable to determine the correct field boundary in the digitization). The ‘no area’ flag is used to (a) remove those fields from any subsequent field area analysis, and (b) alert downstream processing that the polygon does not represent the true boundary. As an example, this situation could occur if several small fields were clustered together, in which case it can be more practical to treat the grouping as a single polygon and express the BA as a percentage of this larger polygon.

### 2.2. Effective BA per fire pixel (α_L_ and α_H_)

These highly detailed mapped reference areas of burned versus unburned cropland fields (42 958 fields in total) were used to calculate the effective BA per fire pixel (*α*) for two time periods (January–June and July–December). As in previous studies [33], the parameter *α* is in effect a conversion factor that can be used to extrapolate our reference areas to much larger regions. Because our high resolution reference areas include an indeterminate label (class 3) for fields that could not be unambiguously labeled as burned or unburned, we calculated lower (*α*_L_) and upper (*α*_H_) limits for *α* which treated (a) only the fields with definitive burns (class 1 and class 2) as actually having burned, and (b) the definite burned fields (class 1 and class 2) as well as the ambiguous fields (class 3) as actually having burned, respectively. Each burned field’s area (classes 1, 2, and 3) was weighted by its BA fraction, summed, and then divided by the total number of filtered VIIRS active fire points within the spatial and temporal constraints of each reference area (equation (1)). The *α*_L_ and *α*_H_ conversion factors, along with the number of filtered VIIRS active fires, for all seven reference areas are shown in table 2.

(1) $$\alpha =\frac{\sum \left(\text{Field Area }\times \text{ Fraction of Field Burned}\right)}{\text{Number of VIIRS Active Fire Points}}.$$

While each seasonal time period (spring and summer) had a range of *α*_L_ and *α*_H_ values (table 2), the within-season variability in the individual *α*_L_ and *α*_H_ values were relatively low. Therefore, we calculated seasonal average *α*_L_ and *α*_H_ values (±1 standard deviation (SD) as an uncertainty) and applied them to the appropriate monthly filtered active fire counts to calculate the effective cropland BA within Ukraine (table 3).

### 2.3. Effective BA: monthly VIIRS active fires

Conversion of the filtered cropland VIIRS active fires to estimated BA was undertaken at the oblast level. This spatial unit was chosen as oblasts represent an administrative boundary within Ukraine and are often used as the basis for resource management and policy decisions. Although the analysis was conducted at a monthly time step, the same method applies to any temporal timeframe. Monthly BA per oblast was calculated by multiplying the appropriate seasonal *α*_L_ and *α*_H_ (table 3) to the filtered VIIRS active fire counts (equations (2) and (3))

(2) $$\text{A}\left(o,t\right)={\text{N}}_{\text{f}}\times \alpha_{\text{L}}$$

(3) $$\text{A}\left(o,t\right)={\text{N}}_{\text{f}}\times \alpha_{\text{H}}$$

where, *A*(*o,t*) is the BA per oblast (*o*) during month *t, N*_f_ is the monthly filtered VIIRS active fire count within the Oblast, and *α*_L_ and *α*_H_ are the average seasonal minimum and maximum effective BA per fire pixel conversation factors.

Sensitivity analyses were performed to determine how the estimated BA would change if these seasonal average conversion factors were only applied to the peak burning months (March and April = spring, and July–October = summer) as opposed to including all months within the analysis (January–June = spring and July–December = summer). With only a 2%–4% difference in total cropland BA, we opted to include all months as the burning patterns changed slightly between 2016 and 2017 (supplementary text S4.0, tables S3(a) and (b)).

Although the reference areas only mapped cropland regions within a small subset of oblasts, the low within-season variability of the individual reference area *α*_L_ and *α*_H_ values and the similar seasonal majority crop types associated with VIIRS active fires (supplementary tables S3(a) and (b)) meant the seasonal spring and summer conversion factors were applied to the appropriate monthly filtered VIIRS active fires over all oblasts.

## 3. Results

Ukraine has two distinct burning peaks throughout the year that follow the crop harvest/planting cycles (supplementary figure S11). Typically the early peak in fire activity occurs in March and April and is primarily associated with preparing fields for planting either maize/corn or sunflower. During 2016 and 2017, cropland fire activity typically began building in the first few days of March after the spring snowmelt, peaking in late March-early April, with the majority of springtime burning occurring in the central and northern oblasts (administrative regions). Specifically, in 2017, there was a distinct concentration (and overall increase) in fire activity in the north-eastern oblasts in the first half of March, followed by large numbers of fires spreading throughout Ukraine with a concentration over the central and northern oblasts in April. The second, larger peak in fire activity occurs in July and August and is primarily associated with the winter wheat harvest in the southern regions of Ukraine. In both 2016 and 2017, burning in early to mid-July begins in Odes’ka (south-west oblast; supplementary figure S9) and spreads towards the eastern Oblasts by late July. As the burning season continues into August, the fire activity begins spreading into the north-western oblasts. The fires in July and August are predominantly associated with the winter wheat harvest, and while the fire activity continued to occur (to a lesser extent) into September and October, these fires were mainly associated with a combination of winter wheat and maize, depending on the Oblast.

Understanding this distinctive fire pattern and its relation to specific crop type planting/harvest cycles will help prioritize the appropriate resources and focus policies in the correct locations. We estimated monthly cropland BA for each oblast in 2016 and 2017 to further understand the spatio-temporal patterns of cropland burning within Ukraine (supplementary tables S3 and S4). Specifically, these results highlight the regions with the greatest need for policy implementation and resource management. The five oblasts with the highest combined 2016 and 2017 total BA and active fire pixels are all located in the southern regions of Ukraine and contain predominantly sunflower and winter wheat crops: Odes’ka, Donets’ka, Zaporiz’ka, Dnipropetrovs’ka, and Khersons’ka oblasts (supplementary figure S9). These five oblasts account for approximately 50% of the total cropland BA and also contain some of the highest wheat production values [35] in the country making these regions a potential source for biofuel initiatives [36, 37].

**Figure 4.**
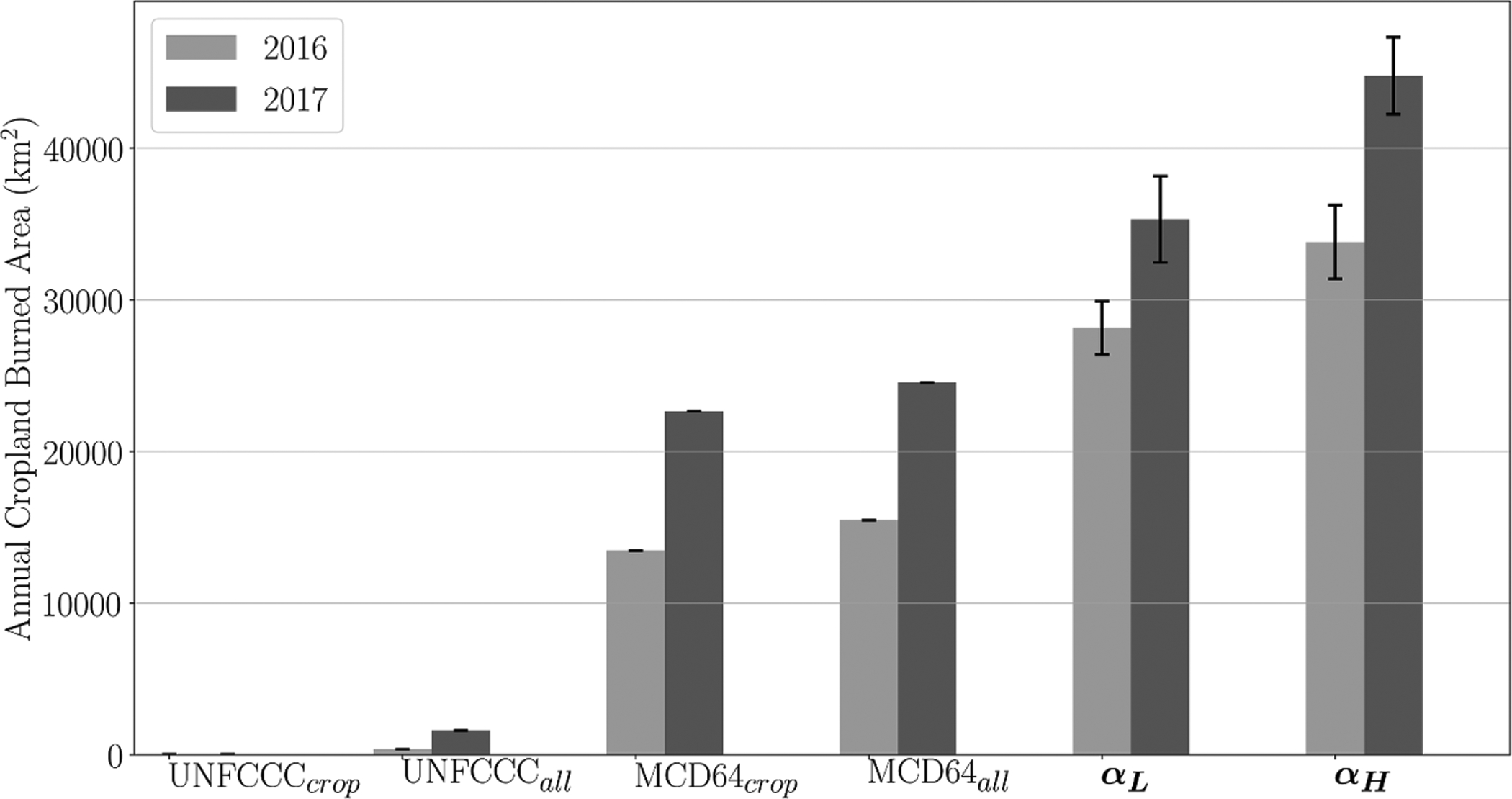
Annual 2016 and 2017 cropland BA (km^2^): (a) UNFCCC cropland estimates [11]; (b) UNFCCC all BA in Ukraine (forest BA, cropland BA, and pastures and wetland BA [11]); (c) MODIS MCD64A1 BA filtered by the MCD12Q1 IGBP Collection 6 [34] cropland and cropland/natural vegetation mosaic classes; (d) MODIS MCD64A1 all BA pixels regardless of land cover type [38]; (e) minimum and maximum effective annual BA calculated using the seasonal average *α*_L_ and *α*_H_ conversion factors, respectively, with error bars of *±*1 SD (see table 3).

To further understand the full extent of burning within Ukraine’s cropland and the magnitude of underestimation in conventional estimates, we compared our estimates of total annual cropland BA in Ukraine with those of the UNFCCC [11] and as reported in the Collection 6 MODIS MCD64A1 BA product [38] (figure 4). The MCD64A1 product is the basis of many burned-area-focused research studies. This coarse spatial resolution (500 m) dataset is useful for describing larger wildfires and region-scale BA patterns and is consequently often used as the basis for several wildfire emission inventories. For example, the global fire emission database (GFEDv4) [39] used the Collection 5.1 MCD64A1 product as an input in producing global fire emissions. However, MCD64A1 has also been used for the basis of many cropland-focused BA and emissions studies [e.g. 40, 41] despite several studies—and indeed the MCD64A1 product documentation itself—all noting that the coarse resolution product significantly underestimates cropland BA [26, 29, 38].

As expected, both sources underestimated the amount of BA, with the magnitude of this underestimation varying depending on the year and, of course, over the range of actual area burned encompassed by the spread of the *α*_L_ and *α*_H_ estimates: 30%–63% (MCD64A1 cropland), 24%–57% (MCD64A1 all BA regardless of land cover type), and by 95%–99% (UNFCCC cropland and all fires) (figure 4). This severe underestimation in the BA values—regardless of land cover type—used in an official government document (UNFCCC [11]) specifically designed for policymakers is particularly concerning as it greatly diminishes the extent of open burning while also underrepresenting the contribution of open burning to the overall national greenhouse gas emissions inventory. Clearly, higher accuracy cropland BA estimates are needed, ideally in conjunction with solutions to reduce prescribed open burning in Ukraine croplands. Interestingly, in our seven mapped areas, 64% of the burned fields belonged to class 2, i.e. those having neither visible flames and/or smoke, nor a proximate VIIRS active fire pixel, but which nevertheless have a distinct BA. This finding not only further confirms some of the challenges associated with mapping cropland burning (e.g. the timing of the burns compared to the satellite overpass times) but also points to the likelihood that our estimates are also underestimated.

A potential solution to reduce open burning in croplands is the incentivization of excess crop residue for the production of biofuel [36, 37, 42]. By conservatively calculating the annual cropland VIIRS fire radiative energy (FRE, units: MJ), we estimated the corresponding biomass consumed as agricultural residue [43, 44]. In this context, FRE can provide a first-order estimate of the amount of potential exploitable energy that is lost through crop residue burning in Ukraine. A conservative lifetime for cropland fires was chosen (1 h) based on timing analysis of burning cropland fields with coincident Planet (morning and afternoon overpasses), Landsat-8, and Sentinel-2 images on the same day as the fire event (supplementary text S5.0). The field burn timing results were confirmed by local expert O. Zhuravel, Food and Agriculture Organization of the United Nations (FAO), Regional Office for Europe and Central Asia. An adjustment factor that compensated for duplicate detections was applied to the filtered cropland VIIRS active fire pixels based on a proximity analysis (supplementary text S5.0). Overall, our estimated annual total FRE associated with cropland burning in Ukraine is 1470 GJ in 2016 and 1710 GJ in 2017. These first-order estimates serve to highlight the importance of continuing research to find sustainable methods to remove excess residue from fields—without depleting too many soil nutrients—as a means of helping achieve Ukraine’s biofuel goals.

## 4. Discussion

Understanding both the magnitude and spatio-temporal patterns of burning in croplands is essential for resource management and policy initiatives. This study not only provides a more realistic understanding of the extent of BA within Ukraine croplands, but it also shows the importance of using the best available data and methods in policy documents. For example, the Ministry of Environment Protection and Natural Resources (MENR) of Ukraine, which is responsible for the creation of Ukraine’s Greenhouse Gas Inventory, is restricted from using remote sensing imagery in their analysis and therefore produces cropland BA estimates based on sporadic and incomplete reports from the State Emergencies Service of Ukraine. Despite significantly underreporting the extent of cropland burning, the satellite-based MCD64A1 BA maps are far more realistic than the BA estimates (cropland and all fires) used within the UNFCCC document. Furthermore, methodologies should also be updated as technology and knowledge improve. For instance, the MENR of Ukraine is constrained by the emissions methodology and definitions set out in the IPCC chapter 2: Generic methodologies applicable to multiple land-use categories section 2.4, equation 2.27 [45]. This generic emissions equation has been found to be inadequate for quantifying crop residue emissions since BA (often severely underestimated) is the primary input variable [e.g. 46, 47]. Finally, this analysis also highlights the importance of including subject and, in particular, local experts in both the creation of policy documents and within scientific studies. There are multiple benefits to including collaborators with different perspectives. Remote sensing experts are able to understand the broad patterns of BA on a national or global scale, whereas local field experts can provide missing and often critical details. In the case of the UNFCCC document, the lack of a remote sensing perspective has led to a severe underestimation in the national emissions inventory.

Ukraine is in the midst of a transitional period encompassing numerous social, economic, environmental, and climatic changes that have heavily impacted both the past and present fire regimes. Since Ukraine’s independence in 1991, drastic changes have occurred in land use/land structure and ownership [48–51]. Specifically, the privatization of 275 000 km^2^ of former Soviet Union kolkhoz lands (state-owned cooperative farm land run by farm laborers) gave 6.92 million citizens a piece of property on average between 0.036 and 0.04 km^2^ (3.6–4.0 ha). As a result, 74.95% of all agricultural lands (total area according to [48] is 414 890 km^2^) became privately owned, 24.06% state-owned, and 0.99% collectively/communally owned. Since 2001, the land market has been banned in Ukraine, leading to the majority of land users (total area of 246 000 km^2^) renting their lands and therefore having no investment in the long term quality of the soil [52]. However, in March 2020, the government moved to lift the ban on the land market in 2021, therefore allowing the sale of farmland [53]. It is expected that the opening of the land market will lead to restructuring of land ownership and ultimately a decrease in the extent of open burning. Specifically, the poorest agricultural producers (0.1–0.15 km^2^ on average), whom often cannot afford environmentally clean technologies and thus typically burn their fields, will be able to sell their lands, thereby leading to an increase in the average area owned by land-users that can afford to invest in new machinery.

Although this is a step in the right direction, the lack of financial and technical support for farmers and land-users who do not want to sell their lands from the government and private industry will continue to further exacerbate open burning in Ukraine. Specifically, the absence of an effective, national-scale, financial incentivization program to help discourage farmers from burning crop residue and instead adopt more environmentally sound field-clearing technologies will likely continue to be a hindrance in the quest for reducing open burning. Fortunately, a new law aimed at reducing this practice was passed in Ukraine in April 2020 [54] following a recent series of large wildfires in Ukraine inadvertently started from cropland burning, including a 1000 km^2^ fire in Zhytomyr and the Chernobyl Exclusion Zone, and a 50 km^2^ fire in the Lugansk oblast (far eastern region of Ukraine) with five fatalities and up to 40 people injured. These large fires attracted the attention of authorities to this often overlooked problem. While the new law has increased fines for open burning to more than 20 times from the previous fines, it is unclear if these expanded penalties will be effective in the reduction of a culturally-ingrained practice.

### 4.1. Cropland BA mapping limitations

Open burning in Ukrainian agricultural lands spans beyond sown cropped fields (325 540 km^2^). Other agricultural lands are also subject to both intentional and unintentional burning, including abandoned lands (2290 km^2^), hay (23 990 km^2^), pasture (54 210 km^2^), and almost all small, private fields (vegetable gardens) near villages [48]. Due to the wide range of fire types, sizes, and intensities within such a heterogeneous landscape, BA estimates are often underestimated. Earlier assessments have shown a wide range of annual BA estimates ranging from 12 800 km^2^ (2010) to 52 700 km^2^ (2015) for all land cover types in Ukraine [55]. This study further highlights some of the limitations of mapping cropland BA using traditional remote sensing approaches. The transient nature of cropland fires and the rapid changes in land cover from harvested to burned to plowed (typically, fields in Ukraine are plowed within 2 d of burning; pers. comm. O Zhuravel, FAO) requires at least a daily, high-resolution image to help capture the field burning before plowing.

At present, daily Planet imagery is available (since *~*2016). However, these data are only acquired in the visible and near-infrared wavelengths and therefore identifying active burns through short-wave infrared imagery is not possible. While Landsat-8 and Sentinel-2 are alternate options, the ~3–5 d overpass (when considering all sensors) leads to an increase in the misinterpretation of burned and plowed versus plowed-only fields, and many of the more subtle changes caused by burning are also indistinguishable at 20 and 30 m resolution. Finally, while coarse-resolution sensors such as MODIS and VIIRS are currently the best options for identifying active fires in Ukraine, many burned fields will have no proximate coarse-resolution active fire pixels (see section 3). Consequently, while our method accounts for fields that were burned but not associated with any flame or active fire point, our estimates may still be lower than the true values. Nevertheless, comparing our BA estimates against the widely used MODIS MCD64A1 BA product, we found that MCD64A1 underestimates cropland BA in Ukraine by 49%–63% in 2016 and 30%–52% in 2017 when non-cropland pixels (IGBP) are excluded, and between 41%–57% in 2016 and 24%–48% in 2017 with non-cropland pixels included (note that these values do not discount partially compensating product commission errors associated with the larger pixel sizes and the known harvest-signal confusion [26, 38]). This underestimation has important implications for fire-based emission inventories, such as the GFEDv4 [39]. Specifically, while these global inventories generally underestimate the small fire contribution within most ecosystems, cropland areas often suffer the greatest proportionate loss since the majority of fires in this land cover class are small [e.g. 27, 56].

### 4.2. Cropland burning: environmental and political implications

Accurately quantifying the timing and extent of open burning in croplands is crucial because these fires, while individually small, collectively have far-reaching impacts beyond their field boundaries. By the middle of the 21st century, Ukraine is expected to see warming across all months, with an average temperature increase of 1.2 °C–1.5 °C across the whole country and a decrease in summer precipitation across *~*80% of the country [57]. The 2010 heat waves resulted in a large number of fires, extremely poor air quality, and reduced net primary production in Eastern Europe and European Russia [58, 59]. A peak period for crop-residue burning in Ukraine occurs in July and August, therefore it is essential to start reducing the potential for an increase in uncontrollable cropland fires in a future drier climate. For example, in some Eastern and Southeastern European countries, which neighbor Ukraine and the EU, excessive agricultural burning constitutes a major reason for the impoverishment of agricultural lands and are a source of uncontrolled wildfires which spread into adjacent forests and protected areas [60]. In most of these countries, laws ban agricultural burning. In practice, however, law enforcement is either insufficient or counterproductive. The latter refers to the practice of farmers and shepherds who continue to not only practice burning cropland fields and pastures but also disappear after ignition in order to avoid penalties leading to uncontrolled wildfires.

Despite the enormity of the task at hand, the goal of reducing open burning is achievable. Examples from the South Caucasus show the intent to regulate agricultural burning through a burning permit system as an interim solution to favor the replacement of open burning practices by alternative methods such as tilling or organic farming [61, 62]. Additionally, Germany serves as an example of a European country that successfully regulated open burning through a variety of legal instruments. Until the early 1970s, burning of crop residues, fallow lands and embankments along roads or between agricultural plots were quite common. Excessive burning activities in the agricultural lands of Germany, which in the 1960s and 1970s became highly mechanized and treated with fertilizers and pesticides, threatened important refugia for endangered flora and fauna [63, 64]. In the 1970s, two federal laws provided the legal framework for developing relevant laws in the 11 states (after unification in 1990 adopted by additional five states): the Federal Emissions Law (1974) and the Federal Conservation Law (1976). In addition, State Forest Laws regulate the use of fire inside and nearby forests. According to these laws, the burning of agricultural residues, fallow lands, and any other open burning is forbidden. However, all legal instruments allow exemptions for prescribed burning, which are often necessary from the point of view of conservation goals and/or otherwise are not detrimental to the vegetation or environment, to air quality, or to human health. Consequently, open burning in croplands have been halted more or less completely.

## 5. Conclusion

The first-step in tackling open agricultural burning in Ukraine requires understanding the extent of the problem. Remote sensing technology allows us to comprehend the magnitude of the burning through an impartial viewpoint and helps remove the ‘out of sight, out of mind’ philosophy that seems to be prevalent given the illegality of open burning. Bringing the attention of these results to the Working Group on Fires within the Parliament of Ukraine (co-authors: Dr S Zibstev and Dr J Goldammer) has helped provide evidence to support the development of a new national strategy for landscape fire management. Actionable steps are required to start moving Ukraine toward reducing cropland burning, for example, through the development of extension services for sustainable agricultural practices or the creation of viable markets for the commercialization of crop residue for bioenergy [65].

## Supplementary Material

Supplementary

## Figures and Tables

**Table 1.** Summary information on the seven reference areas used in the BA mapping analysis. Locations of reference areas are shown in figure 3.

Reference area	Mapping start date	Mapping end date	Predominant crop type	Mapped area (km^2^)	Cropland area (km^2^)	Cropland fields classified
A	1 March 2017	31 March 2017	Maize/sunflower	1998	1499	3998
B	1 March 2017	31 March 2017	Maize	6021	3557	6168
C	1 July 2017	4 August 2017	Winter wheat	6136	3832	9306
D	1 August 2016	31 August 2016	Winter wheat/maize	3367	2511	5089
E	15 July 2017	15 August 2017	Winter wheat/maize	4302	2474	5440
F	1 June 2017	27 July 2017	Winter wheat	2318	1297	2760
G	15 June 2017	31 July 2017	Winter wheat	4587	2813	10 197
Total				28 729	17 983	42 958

**Table 2.** Low (*α*_L_) and high (*α*_H_) conversion factors for all seven reference areas (A–G). The low conversion factor (*α*_L_) represents the effective BA per fire pixel when only including the fields with definitive burns (class 1 and class 2), whereas, the high conversion factor (*α*_H_) includes both the definite burned fields and the ambiguous fields (class 3).

Reference area	Season	*α*_L_	*α*_H_	Filtered VIIRS active fire counts
A	Spring	0.49	0.91	87
B	Spring	0.68	0.92	392
C	Summer	0.51	0.55	805
D	Summer	0.52	0.64	498
E	Summer	0.48	0.58	348
F	Summer	0.47	0.53	307
G	Summer	0.46	0.50	1511

**Table 3.** Seasonal average and SD low (*α*_L_) and high (*α*_H_) effective BA per fire pixel conversion factors. The low conversion factor (*α*_L_) represents the effective BA per fire pixel when only including the fields with definitive burns (class 1 and class 2), whereas, the high conversion factor (*α*_H_) includes both the definite burned fields and the ambiguous fields (class 3).

Season	Average *α*_L_	SD *α*_L_	Average *α*_H_	SD *α*_H_
Spring (January-June)	0.59	0.1	0.92	0.01
Summer (July-December)	0.49	0.02	0.56	0.05
