# Supplementary material for: Environmental and political implications of underestimated cropland burning in Ukraine
Source: Environ Res Lett. Author manuscript; Available in PMC 2021 Jul 26. (PMC8312694; doi:10.1088/1748-9326/abfc04)
Supplement: Supplementary [file NIHMS1710350-supplement-Supplementary.docx]

**Environmental and Political Implications of Underestimated Cropland Burning in Ukraine**

**Supplementary Information Text**

**S1.0 – Data: Land Cover Classification Maps**

The map creators used freely available satellite images [1-3], well-established image processing methods [4-8], and recommended practices within the Joint Experiment for Crop Assessment and Monitoring (JECAM) [9, 10] to map land and crop types in Ukraine in 2016 and 2017. Specifically, they used all available data acquired by Landsat-8, Sentinel-2, and Sentinel-1 satellites to generate time-series of surface reflectance for optical and backscatter coefficients for radar data to be used as input features for land cover classification and mapping. They used a deep learning approach [5], which consists of an ensemble of neural networks. Multilayer perceptron (MLPs) is the main component used for per-pixel classification. Multiple MLPs were trained using different parameters and architectures (number of hidden layers) and formed an ensemble. Each MLP had a rectified linear unit (ReLU) function as the activation function for neurons in the hidden layers, and training of the MLPs was performed using a stochastic gradient-based optimizer [11]. They used L2 regularization (with coefficient 0.1) to prevent MLPs from overfitting. Learning rate was set to 10^-3^. A separate MLP ensemble was used for crop classification and land cover maps for Ukraine in 2016 and 2017 using Landsat-8, Sentinel-1 and Sentinel-2 imagery and corresponding labelled data collected through extensive ground surveys. The spatial resolution of the resulting maps was 10 m. Overall, accuracies for major crop types, such as winter cereals, winter rapeseed, maize, soybeans, and sunflower range from 85% to 95%.

**S2.0 – Data & Methods: S-NPP Visible Infrared Imaging Radiometer Suite (VIIRS) Active Fires Filtering and Verification**

*S2.1 – S-NPP VIIRS Data*

The 2016 and 2017 S-NPP VIIRS (375 m) active fire locations (VNPIMG14ML C1 V2; [12]) were obtained from the University of Maryland’s ftp server (ftp://fuoco.geog.umd.edu; [13]). This version of the S-NPP VIIRS product includes an additional quality check by the product developer to remove bad calibration-maneuver scans missed in the official dataset available from the NASA Atmosphere Archive and Distribution System (LAADS) DAAC (https://ladsweb.modaps.eosdis.nasa.gov/). The active fire locations were buffered using a variable radius – determined from the pixel area attribute (area = π*r*^2^) – to account for the variation in footprint extent across the VIIRS scan. All active fire locations contain a number of attributes, including the latitude, longitude, date, and UTC time to the nearest minute.

*S2.2 S-NPP VIIRS Active Fires Cropland Filtering*

To determine the land cover type associated with each buffered active fire polygon, we extracted the land cover/crop-type classification pixels for each category (see Table S1) and converted these into a percentage of the total land cover pixels within each active fire polygon boundary. These percentage values were used to exclude active fires associated with urban, forest, and grassland classes. We performed a sensitivity analysis to determine appropriate percentage thresholds for each of these three classes to eliminate an active fire pixel from further consideration as a cropland fire. For this analysis, an active fire polygon was removed if it contained: i) 30% or more urban pixels, ii) 60% or more forest pixels, iii) 50% or more grassland pixels, iv) 75% or more urban, grassland, and forest pixels combined AND cropland less than 10%, or v) less than 10% cropland.

We chose a conservative threshold for the percentage of urban pixels within the active fire polygon to i) reduce the likelihood of including industrial fires, and ii) to reduce the potential of including incorrectly mapped fires (e.g. false alarms caused by bright rooftops; [12]) from the original product. The forest cover threshold was chosen at a less conservative 60%. This threshold allowed for fire polygons with majorly forest pixels to be excluded but allowed some fires to be included that occur either along forest edges or that include a portion of the neighboring shelterbelts or small forested areas that occur throughout the cropland area in Ukraine.

A minimum grassland threshold of 50% was chosen to ensure only fires with a majority grassland land cover were removed, allowing for the inclusion of fires that may contain smaller percentages of grassland that occur between cropland fields. A combined threshold of 75% was also included as initial tests found several fires in January and February (outside of the typical cropland burning window) to contain slightly under the threshold cutoff for the urban, grassland, and forest categories but also contained no cropland classes. By adding the AND into the fourth threshold, we did not remove fires which may have contained 75% grassland, urban, and grassland but between 10 and 25% cropland. The months of March, April, July, August, and September (typical burning months) had the largest absolute number of active fires removed from the analysis. As a relative percentage, however, the months outside of the typical cropland burning window had the highest percentage removed (Figure S1).

*S2.3 Active Fire - Land Cover Verification*

Before the fires were used in the analysis, a subset of fires were visually assessed against remote sensing imagery by all the co-authors and several analysts to ensure the filtering thresholds were appropriate. Landsat-8 OLI imagery was used to visually identify and confirm the land cover type (cropland or non-cropland) associated with a small subset of the filtered active fire polygons. Fires that occurred on the same day or the day before a Landsat-8 overpass (62 unique path/rows) were extracted to avoid any potential loss of the burned area signal between overpasses. Of 56,461 filtered VIIRS active fires, 30,936 fires occurred on the day or the day before a Landsat-8 image ranging between 1 and 949 active fires, depending on the date and location of the image. Images and their associated fires with a range of geographic locations and dates were selected to ensure a representative sample. From these chosen images, approximately 15% of fires were randomly selected for further analysis, for a total of 855 fire samples.

For this further analysis, an analyst examined a combination of Landsat-8 and/or Sentinel-2 images on the day of the observed active fire to identify the location of the fire or subsequent burned area with respect to the land cover class. For example, if a fire polygon contained primarily winter wheat pixels, the analyst would attempt to confirm that the active fire or subsequent burned area occurred within a cropland field, as opposed to within a potential neighboring grassland (or other land cover type) area. This visual verification helped ascertain whether the above mentioned urban, grassland, and forest thresholds were suitable for filtering out the presumed non-cropland fires. Of the 855 fire samples, 7% were determined not to be associated with cropland. The majority of these occurred in March (33) and April (18) and were associated with the small grassland features/windrows between fields. Finally, due to the general low confidence of cropland burns, no active-fire confidence thresholding was applied to these filtered fires. These final filtered active fire points were used as the basis for the cropland burned area analysis (Table S3a and S3b).

**S3.0 – Regional Mapped Output: Reference Areas**

*S3.1 – Field Burned Area*

Of the 42,958 fields digitized, 7,436 were classified as a Class 1 or 2 (i.e., definitely burned) while 1,321 fields were classified as Class 3 (i.e., ambiguous). Unsurprisingly, the two Spring reference areas (Region A and B) contained the highest number of ambiguous fields compared to the Summer reference areas. For all 7 reference areas, the majority of the Class 3 fields were classified as 100% burned. This was also unsurprising since the most common reason for being classified as a Class 3 is the uncertainty between a field being burned and then plowed or simply plowed. Even with daily overpass of Planet imagery, the conversion from a harvested field to a plowed field can be very brief, leaving analysts unable to ascertain if burning occurred in between the change from a light field to a uniform, very dark field.

Overall, of the 7,436 Class 1 and Class 2 fields, 57% were full-field burns with 100% of the field burned, while 79% of fields had more than 50% of their area burned (Figure S10).

**S4.0 – Burned Area/Active Fire Scaling Methods**

*S4.1 - Monthly Oblast Active Fire: Majority Crop Type*

Conversion of the cropland VIIRS active fires to estimated burned area was undertaken at the Oblast level. This spatial unit was chosen as Oblasts represent an administrative boundary within Ukraine and are often used as the basis for resource management and policy decisions. Although the analysis was conducted at a monthly time step, the same method applies to any temporal timeframe.

The first step was to determine the number of cropland active fires per month per oblast and which majority land cover and crop type was associated with these fires. This not only helped further highlight the spatio-temporal patterns of the cropland burning with respect to the crop type, but also helped guide our scaling analysis (Table S3a and S3b). The burning patterns clearly follow the known crop calendar cycles (https://cropmonitor.org/) with the majority of July – September fires occurring in predominantly winter wheat fields (post-harvest) in the southern regions of Ukraine and the majority of March and April fires occurring in maize and sunflower (pre-planting) fields.

The majority crop type was calculated by first querying the distribution of land cover/crop type pixels within each active fire pixel boundary per month per oblast. The majority crop type was labelled for each fire and the class with the highest count was assigned the majority crop type that burned for that oblast.

Monthly burned area per oblast was calculated by multiplying the appropriate α_L_ and α_H_ to the filtered VIIRS active fire counts (equation S1 and S2).

A(*o,t*) = N*_f_* × α_L_ (S1)

A(*o,t*) = N*_f_* × α_H_ (S2)

Where, A(*o,t*) is the burned area per oblast (*o*) during month *t*, N*_f_* is the monthly filtered VIIRS active fire count within the oblast, and α_L_ and α_H_ are the *effective burned area per fire pixel* conversation factors. This is a similar methodology used within the Global Fire Emission Dataset [14].

Due to the low within-season variability of the individual reference areas (e.g. α_H_ in spring in reference area A was 0.91 and in reference area B was 0.92) we opted to reduce the complexity of the scaling from reference area to the Oblast/country level. If the within-season variability was high and the agricultural practices and majority crop types varied substantially, then the α_L_ and α_H_ values would have been weighted by geographic area, crop type etc. Furthermore, given that the 5 main Oblasts with the highest active fire counts contained 4 of the 7 reference areas, we felt these reference values were applicable to scale up to the country-level.

**S5.0 – Fire Radiative Energy**

*S5.1 – Cropland Field Burning: Complete Field Burn Time*

We analyzed 237 burning fields with coincident Landsat-8, Sentinel-2, and morning and afternoon Planet images to determine the approximate duration of cropland field burns in Ukraine. Of this sample, 43 fields had all four images available allowing us to more accurately estimate the approximate burn times. While the temporal sampling was too course to more precisely estimate the time, the majority of the fires had started and were extinguished within the 6 hours between Planet overpasses and therefore, we were able to apply an upper limit to the cropland burn time.

The burn time is consequently likely overestimated and therefore a more conservative 1-hour estimate was used in the calculation of fire radiative energy (FRE). Out of the 43 fields, 5 fields went from unburned to fully burned in less than 6 hours, 34 fields were burned within 6 hours, and 4 fields were burned within 12 hours. Factors such as wind speed, biomass, number of ignition points, field size, etc. all contribute to the variation in times to burn these fields. Based on discussions with local Ukrainian farmer and coauthor O. Zhuravel, we believe the typical burn time is closer to 1 – 2 hours, thus we chose a conservative estimate of 1 hour to represent the time to fully burn a cropland field in Ukraine. Since over 57% of the burned fields digitized in the seven reference areas were fully burned, and 79% of them were at least 50% burned, using a 1 hour burn time to represent all cropland burning in Ukraine is not unreasonable.

*S5.2 – Proximity Analysis*

An adjustment factor that compensated for duplicate detections was applied to the filtered cropland VIIRS active fire pixels based on a 200 m proximity threshold. A sensitivity analysis was performed to determine the final threshold. Same day filtered VIIRS fire polygons within 50 m – 400 m of another fire polygon were removed from analysis. A distinct change in the number of duplicate observations occurred at 400 m since the distance is larger than the average VIIRS 375 m pixel size. Between 50 m and 350 m, the total annual FRE ranged from 1321 GJ – 1607 GJ in 2016 and 1541 GJ – 1873 GJ in 2017.

*
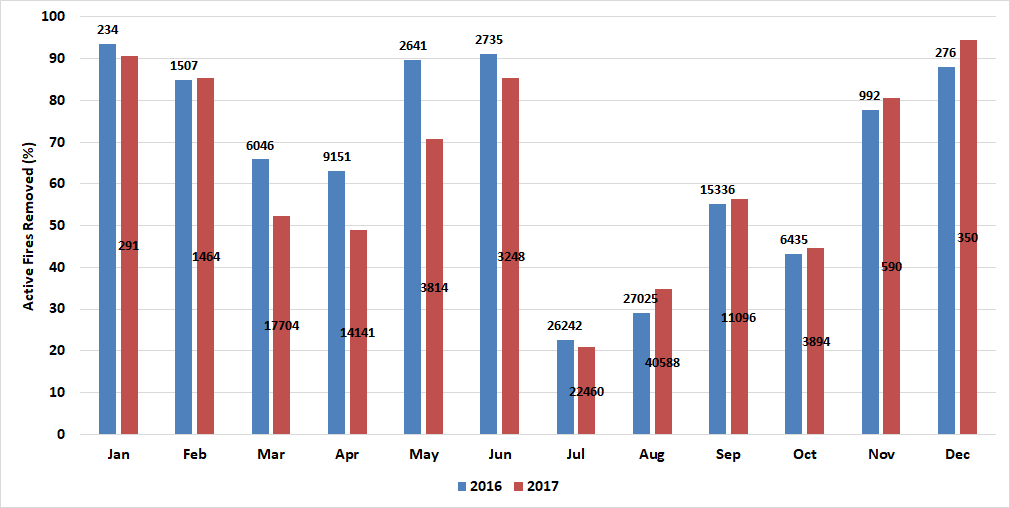
*

Figure S1: Percentage of active fires removed per month in 2016 and 2017 based on the urban, forest, grassland, and cropland percentage thresholds. The values are the original number of VIIRS active fires before filtering.


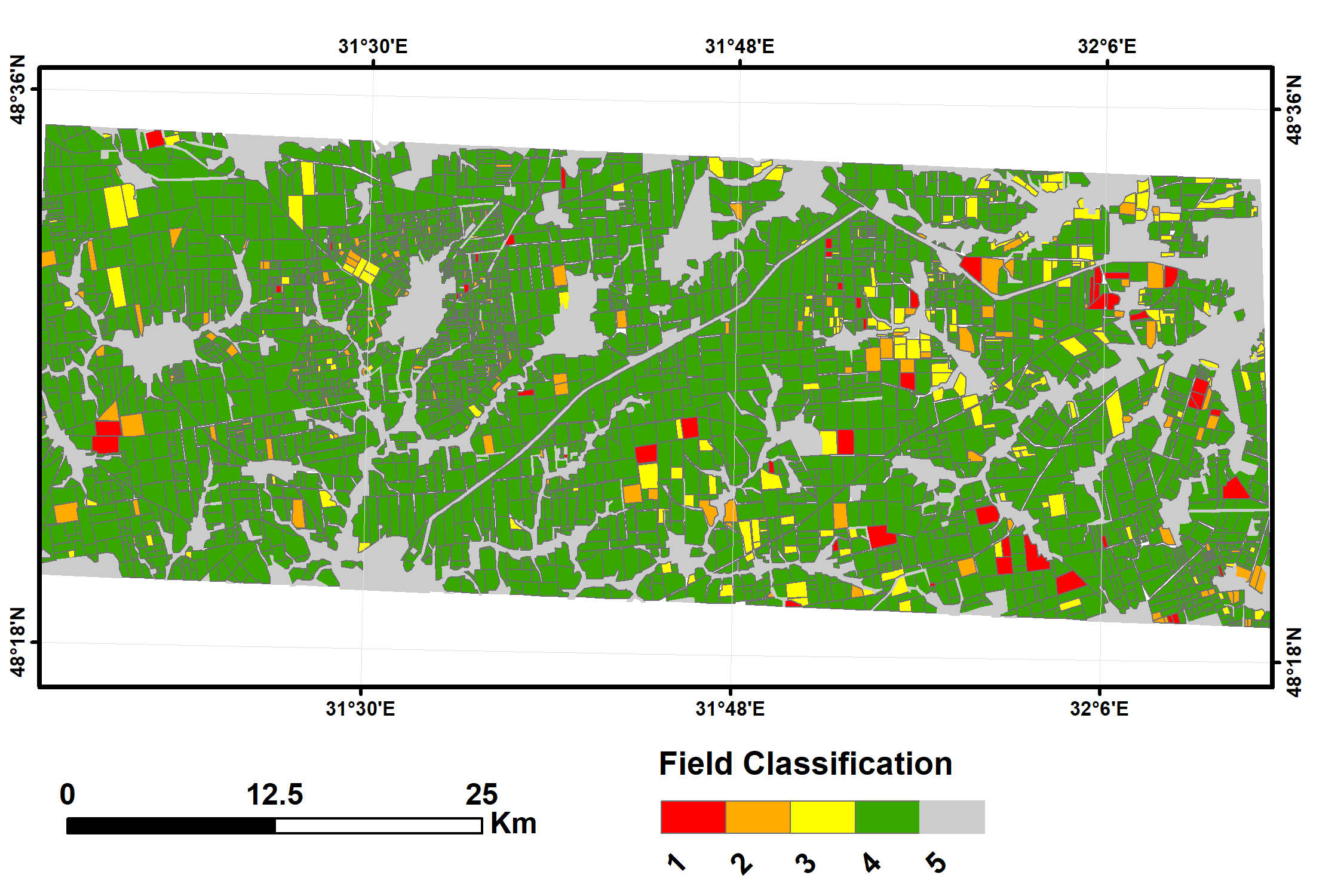


Figure S2 – Reference area A field boundaries and classifications. All polygons were attributed with the following field classification: 1 = active flame or burned area with corresponding VIIRS active fire point; 2 = definite burned area but with no flame or active fire point; 3 = ambiguous (a distinct darkening occurred on the field, but analyst is unsure if the field was burned then plowed or only plowed); 4 = definitely unburned; 5 = non-cropland or fields are too small that land cover conditions were difficult to determine on very high resolution (3 m) imagery.


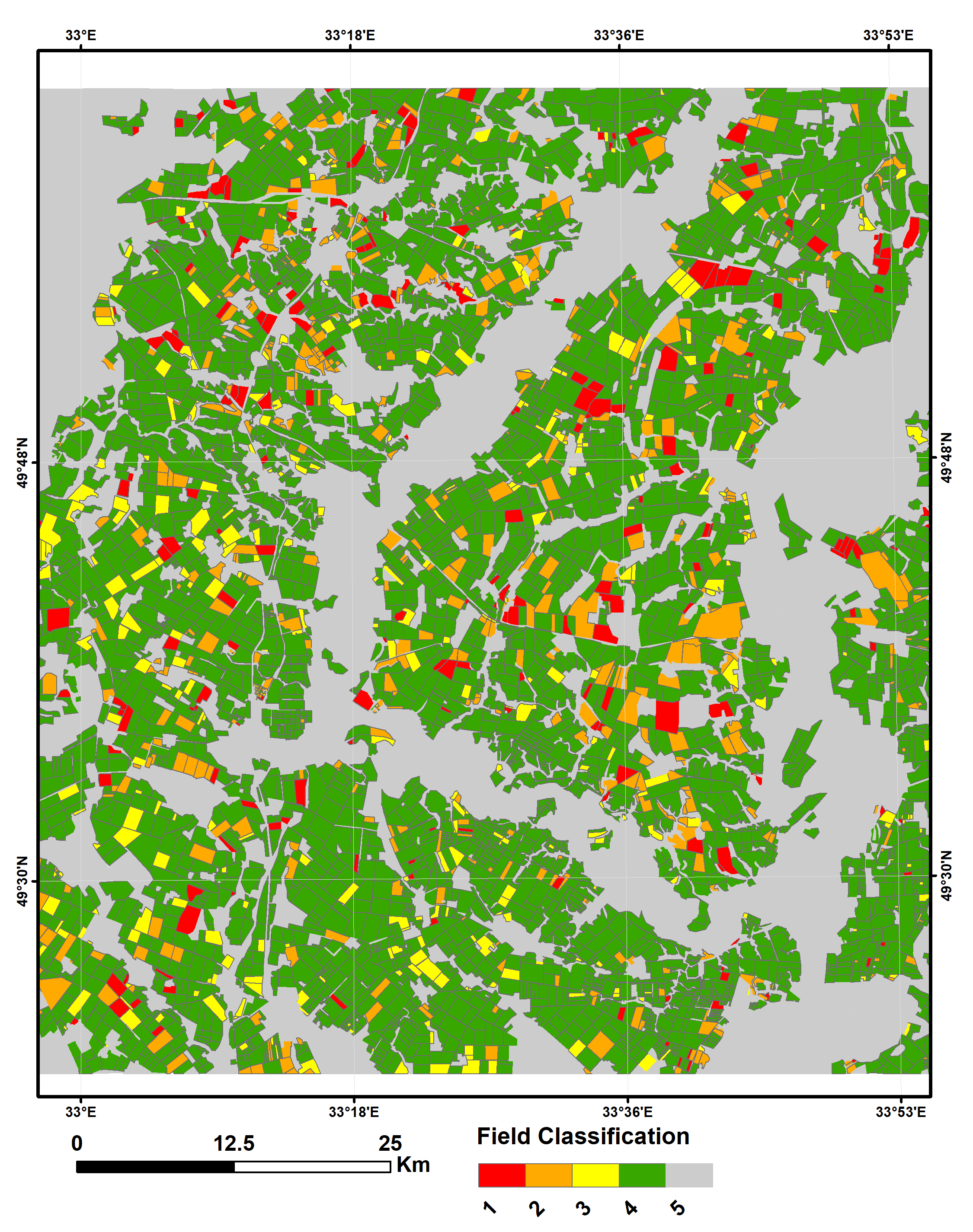


Figure S3 – Reference area B field boundaries and classifications. All polygons were attributed with the following field classification: 1 = active flame or burned area with corresponding VIIRS active fire point; 2 = definite burned area but with no flame or active fire point; 3 = ambiguous (a distinct darkening occurred on the field, but analyst is unsure if the field was burned then plowed or only plowed); 4 = definitely unburned; 5 = non-cropland or fields are too small that land cover conditions were difficult to determine on very high resolution (3 m) imagery.


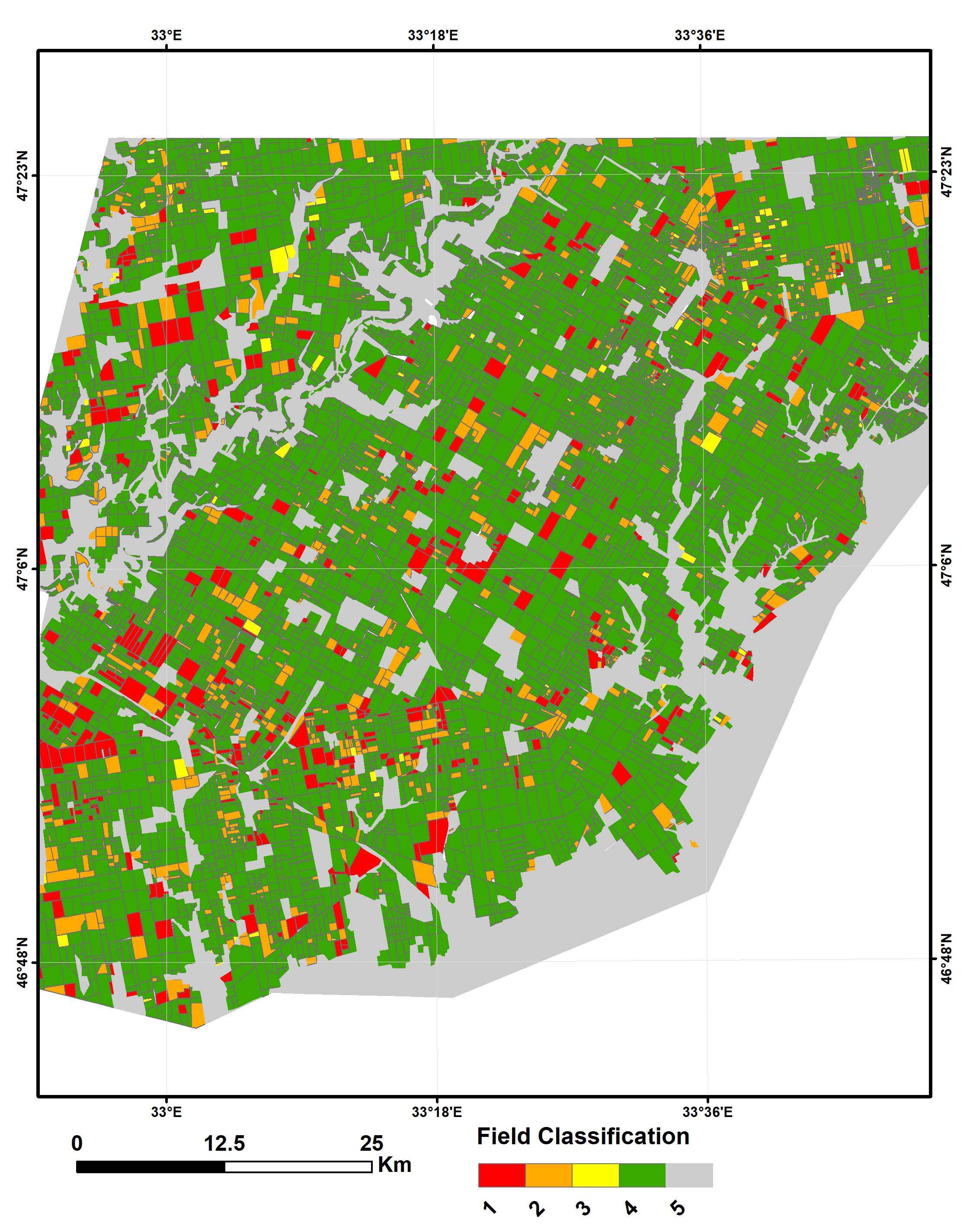


Figure S4 – Reference area C field boundaries and classifications. All polygons were attributed with the following field classification: 1 = active flame or burned area with corresponding VIIRS active fire point; 2 = definite burned area but with no flame or active fire point; 3 = ambiguous (a distinct darkening occurred on the field, but analyst is unsure if the field was burned then plowed or only plowed); 4 = definitely unburned; 5 = non-cropland or fields are too small that land cover conditions were difficult to determine on very high resolution (3 m) imagery.


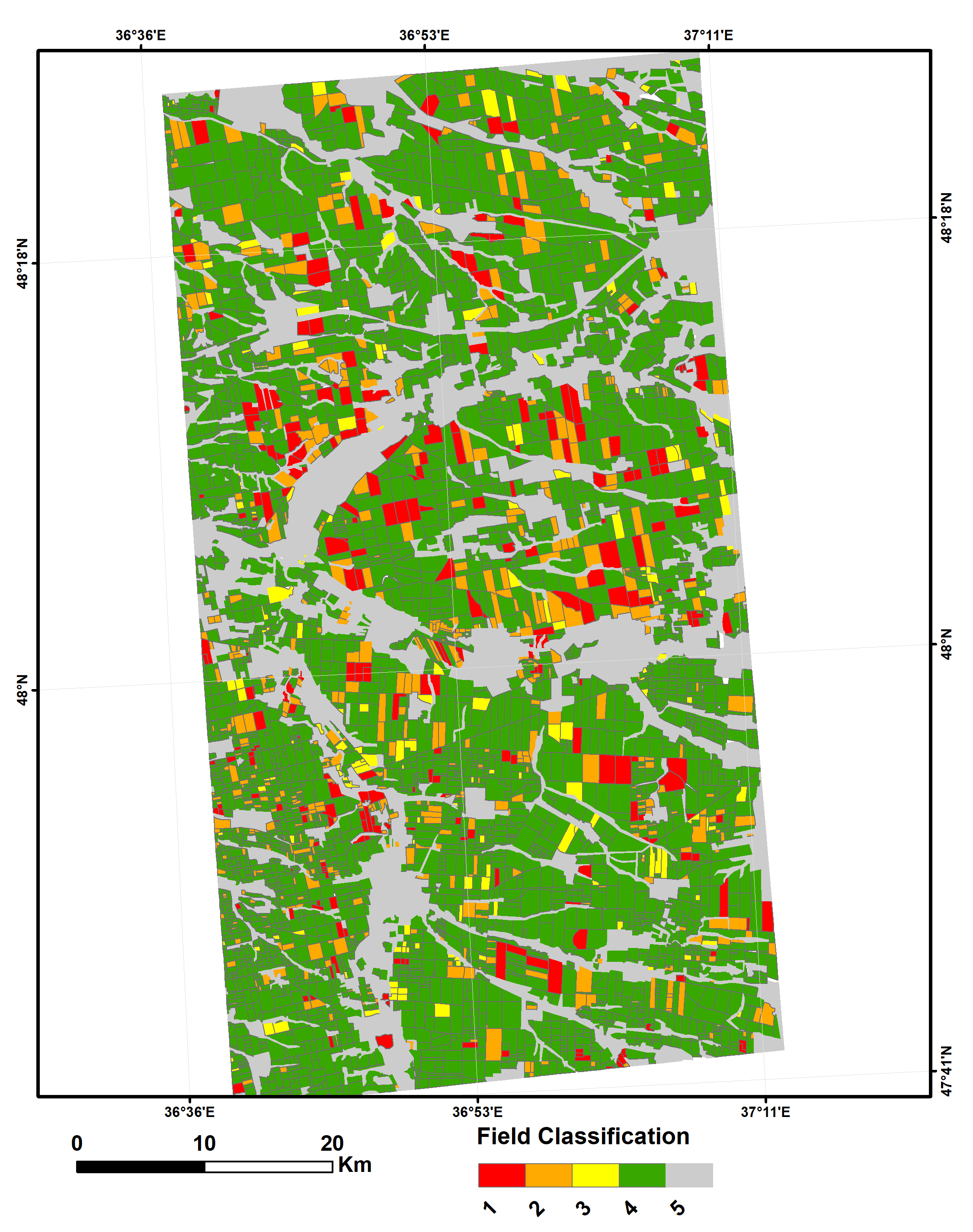


Figure S5 – Reference area D field boundaries and classifications. All polygons were attributed with the following field classification: 1 = active flame or burned area with corresponding VIIRS active fire point; 2 = definite burned area but with no flame or active fire point; 3 = ambiguous (a distinct darkening occurred on the field, but analyst is unsure if the field was burned then plowed or only plowed); 4 = definitely unburned; 5 = non-cropland or fields are too small that land cover conditions were difficult to determine on very high resolution (3 m) imagery.


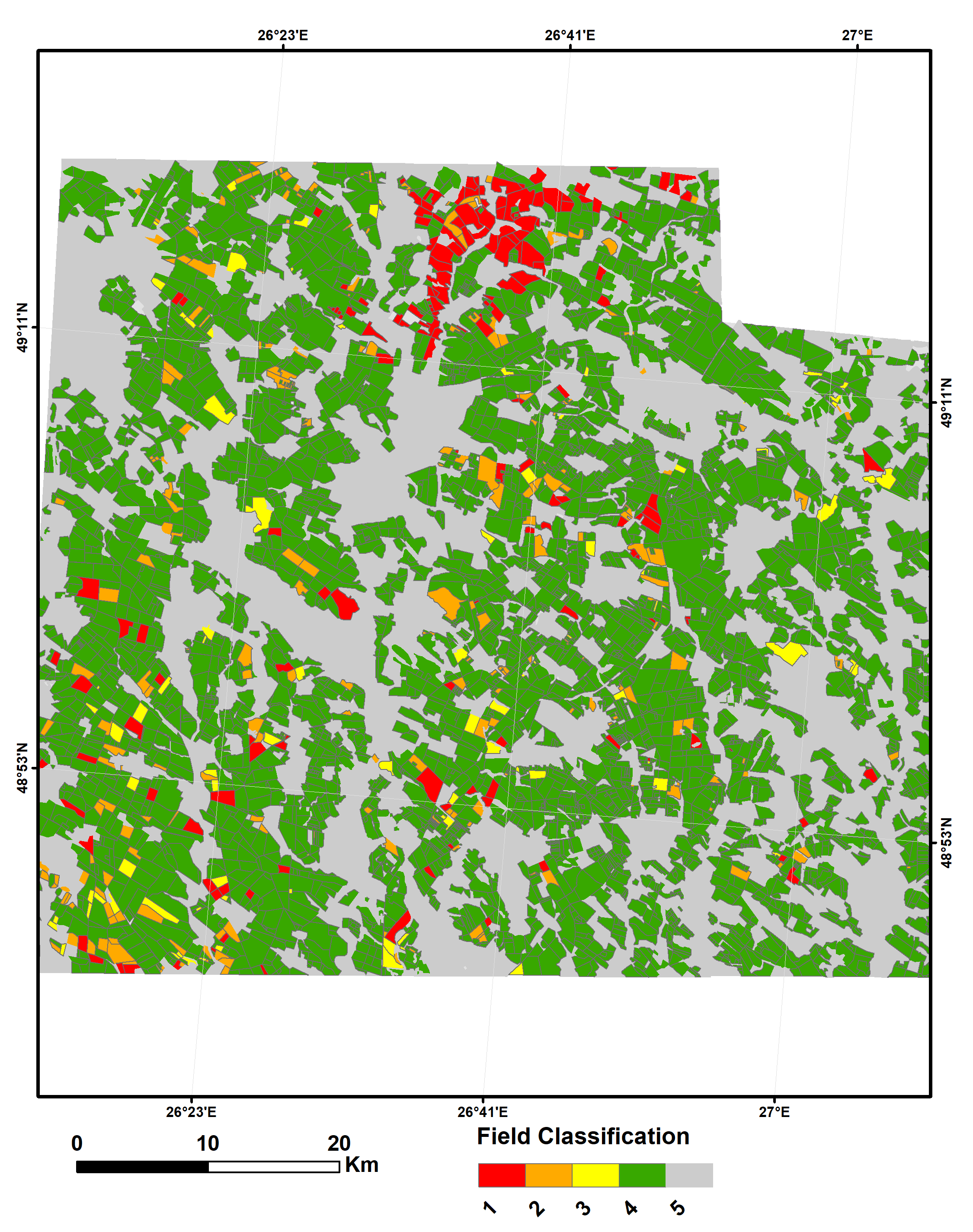


Figure S6 – Reference area E field boundaries and classifications. All polygons were attributed with the following field classification: 1 = active flame or burned area with corresponding VIIRS active fire point; 2 = definite burned area but with no flame or active fire point; 3 = ambiguous (a distinct darkening occurred on the field, but analyst is unsure if the field was burned then plowed or only plowed); 4 = definitely unburned; 5 = non-cropland or fields are too small that land cover conditions were difficult to determine on very high resolution (3 m) imagery.


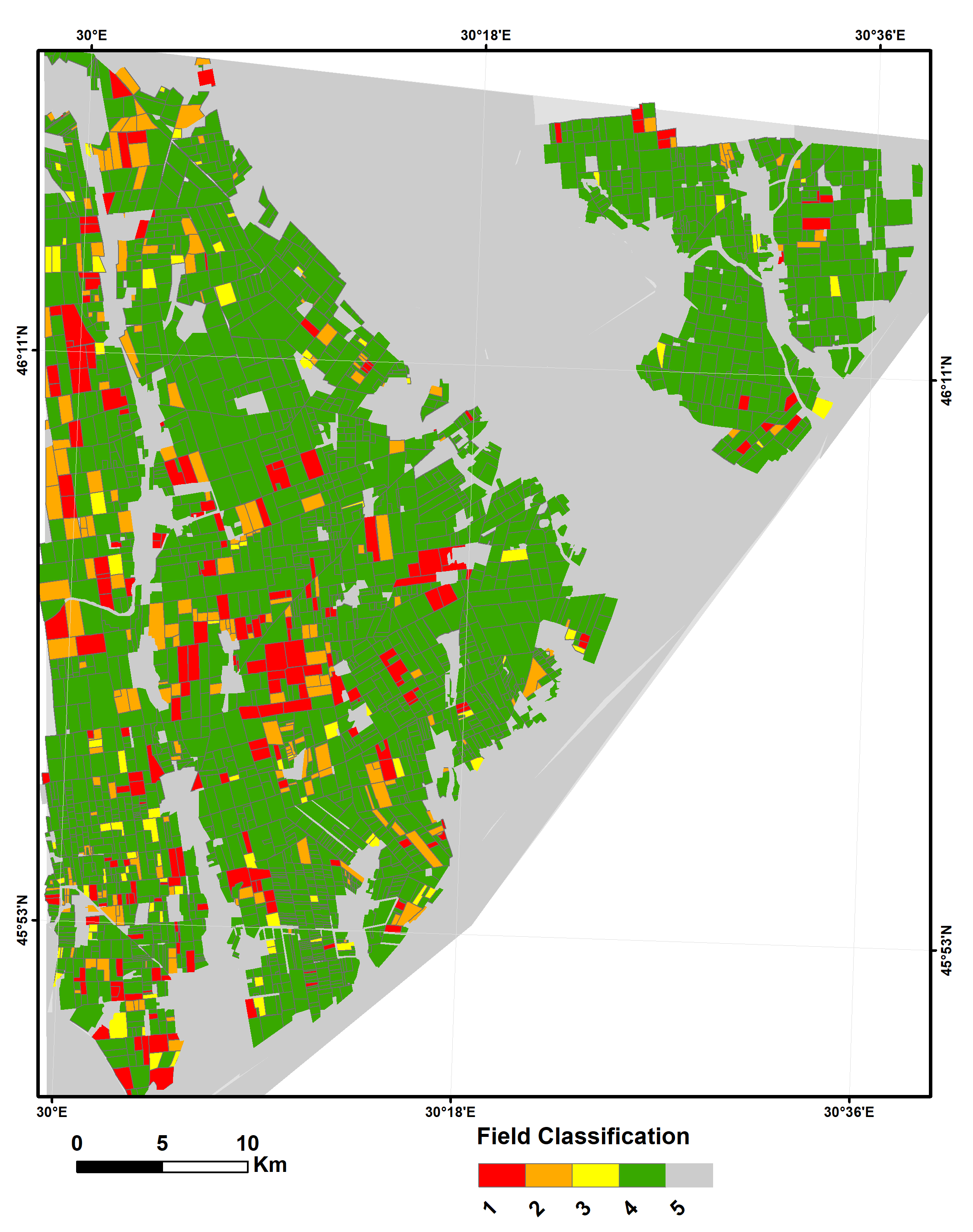


Figure S7 – Reference area F field boundaries and classifications. All polygons were attributed with the following field classification: 1 = active flame or burned area with corresponding VIIRS active fire point; 2 = definite burned area but with no flame or active fire point; 3 = ambiguous (a distinct darkening occurred on the field, but analyst is unsure if the field was burned then plowed or only plowed); 4 = definitely unburned; 5 = non-cropland or fields are too small that land cover conditions were difficult to determine on very high resolution (3 m) imagery.


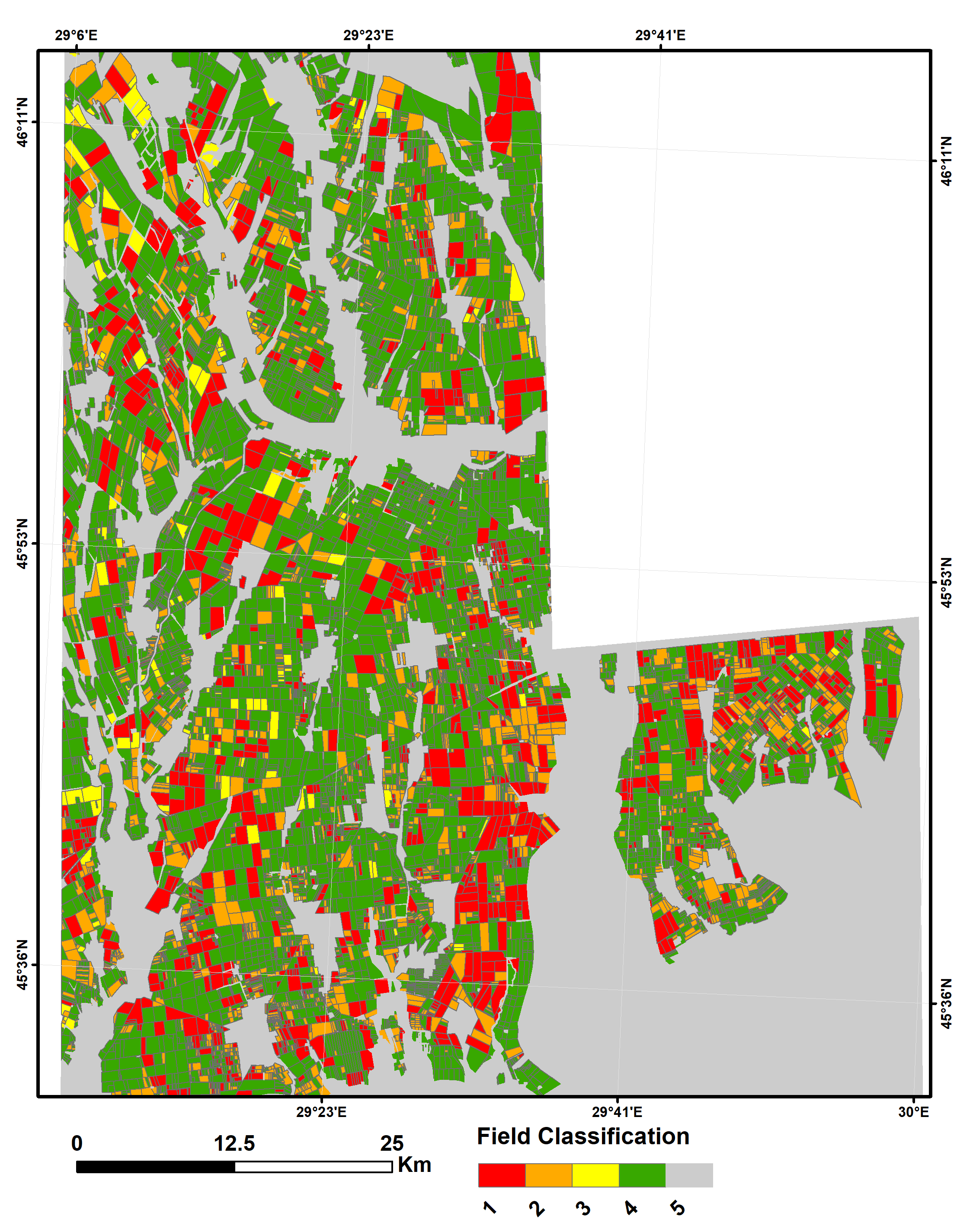


Figure S8 – Reference area G field boundaries and classifications. All polygons were attributed with the following field classification: 1 = active flame or burned area with corresponding VIIRS active fire point; 2 = definite burned area but with no flame or active fire point; 3 = ambiguous (a distinct darkening occurred on the field, but analyst is unsure if the field was burned then plowed or only plowed); 4 = definitely unburned; 5 = non-cropland or fields are too small that land cover conditions were difficult to determine on very high resolution (3 m) imagery.


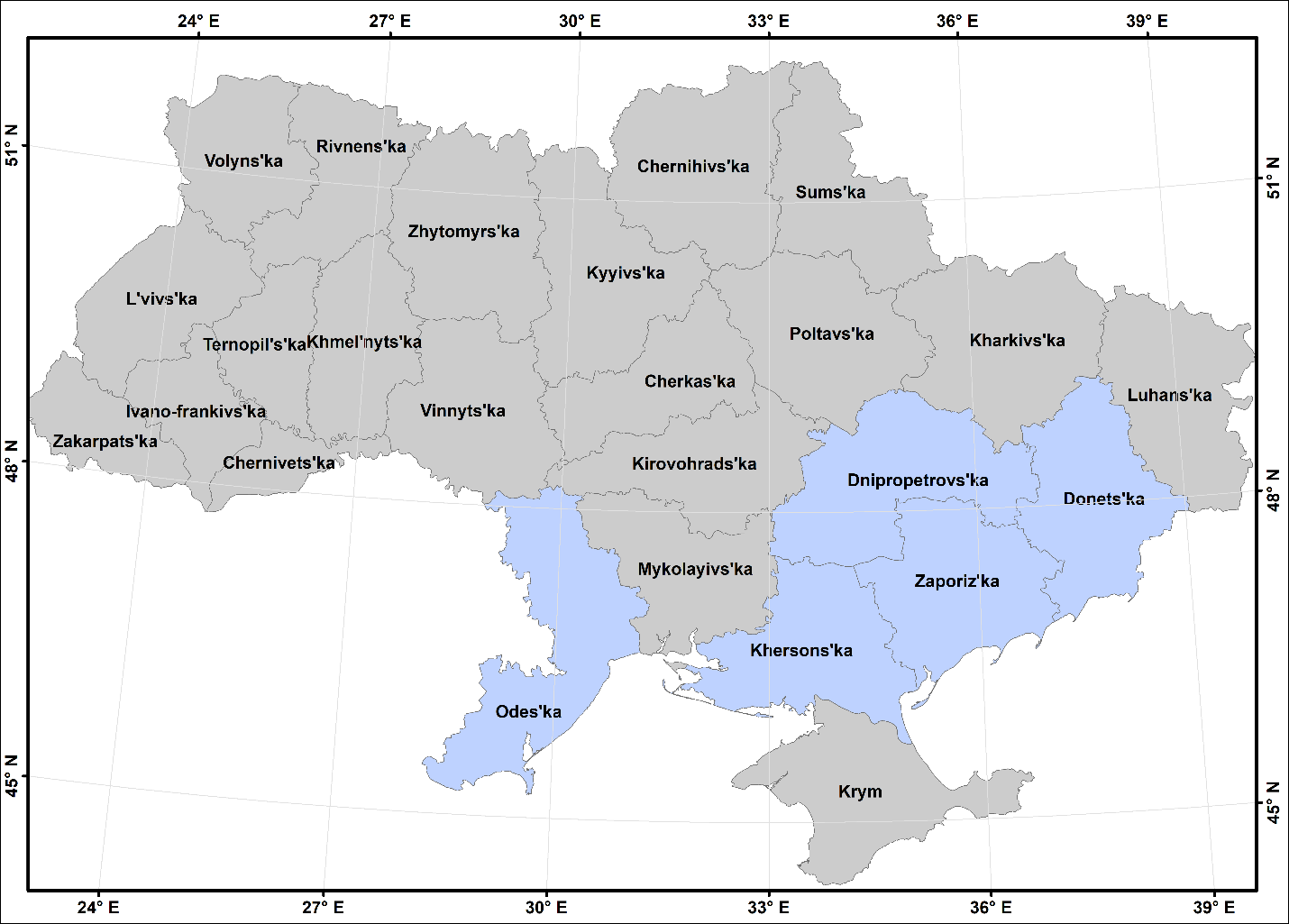


Figure S9 – Ukraine Oblasts. The five Oblasts with the highest combined 2016 and 2017 total burned area and active fire pixels are highlighted in blue.


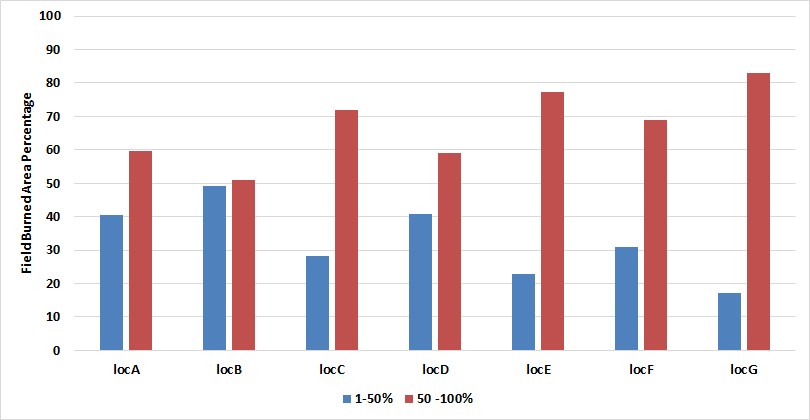


Figure S10 - Class 1 and Class 2 field burned area percentage for each mapped reference area (locA – locG): Class 1 = Active flame or burned area with corresponding VIIRS active fire point and Class 2 = Definite burned area but with no flame or active fire point.


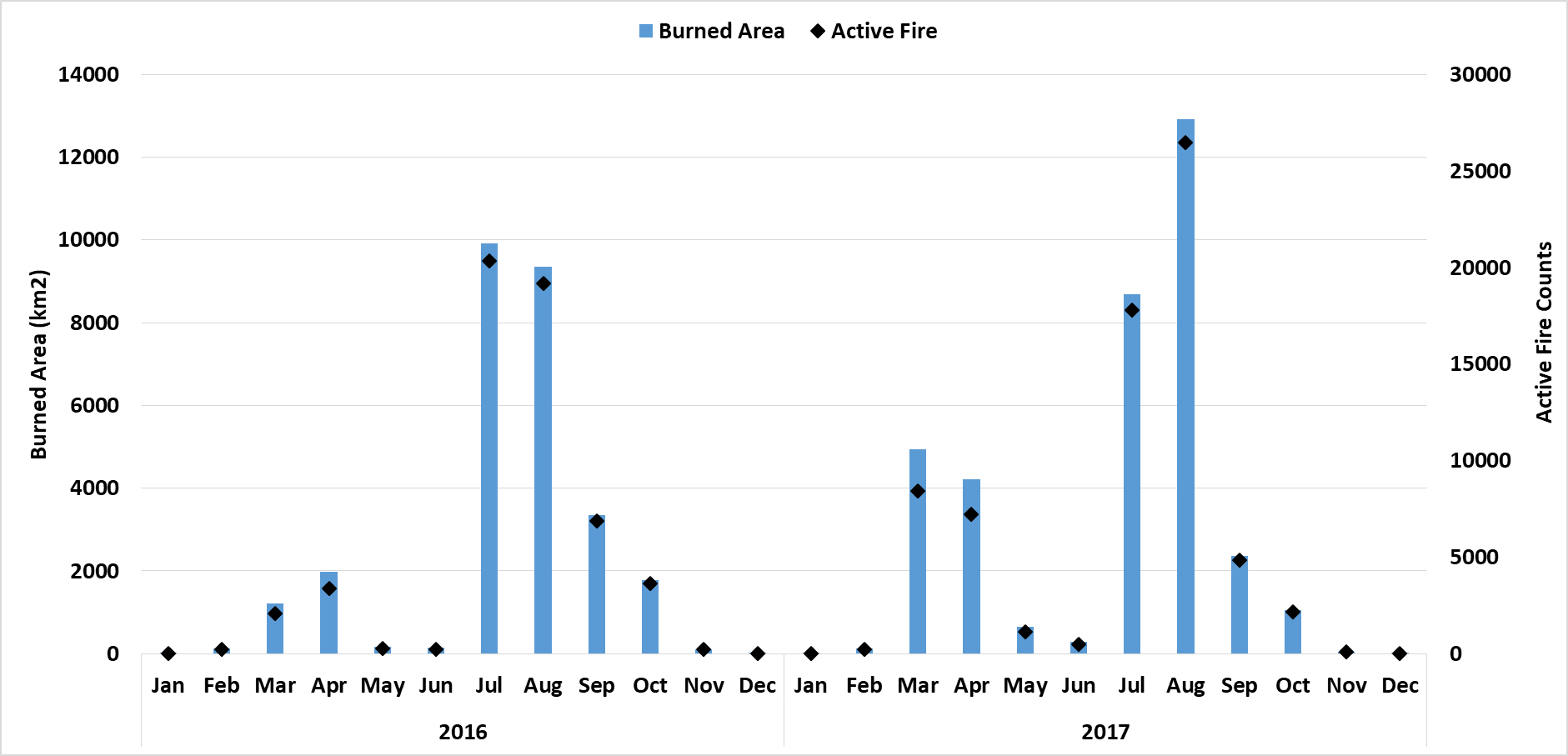


Figure S11 - 2016 and 2017 cropland filtered VIIRS monthly active fire counts (black diamonds) and the minimum burned area estimates using the spring average α_L_ conversion factor (0.59) for VIIRS active fire counts between January and June and the summer average α_L_ conversion (0.49) factor for VIIRS active fire counts between July and December.

**Table S1:** Land Cover and Crop Type classification and corresponding 2016 and 2017 area (km^2^). Burnable crops are highlighted (*)

| Pixel Class | Land Cover/ Crop Type | 2016 area (km^2^) | 2017 area (km^2^) | Area Difference (2017 – 2016; km^2^) |
| --- | --- | --- | --- | --- |
| 1 | Artificial/urban | 14,904 (2.5%) | 18,558 (3.1%) | 3,653 |
| 2 | Winter wheat* | 86,516 (14.3%) | 100,844 (16.7%) | 14,329 |
| 3 | Winter rapeseed | 7,612 (1.3%) | 9,103 (1.5%) | 1,491 |
| 4 | Spring crops (wheat, barely) | 2,546 (0.4%) | 0 | -2,546 |
| 5 | Maize* | 53,683 (8.9%) | 63,972 (10.6%) | 10,289 |
| 6 | Sugar beet | 2,573 (0.4%) | 3,987 (0.7%) | 1,413 |
| 7 | Sunflower | 81,352 (13.5%) | 81,989 (13.6%) | 637 |
| 8 | Soybeans* | 24,917 (4.1%) | 21,537 (3.6%) | -3,381 |
| 9 | Other cereals | 2,861 (0.5%) | 0 | -2,861 |
| 10 | Forest | 155,875 (25.8%) | 149,166 (24.7%) | -6,709 |
| 11 | Grassland | 117,944 (19.5%) | 115,804 (19.2%) | -2,140 |
| 12 | Bare land | 7,871 (1.3%) | 9,695 (1.6%) | 1,824 |
| 13 | Water | 16,448 (2.7%) | 17,097 (2.8%) | 648 |
| 14 | Wetland | 7,790 (1.3%) | 7,531 (1.2%) | -259 |
| 15 | Winter barley* | 17,629 (2.9%) | 0 | -17,629 |
| 16 | Peas | 3,138 (0.5%) | 5,120 (0.8%) | 1,981 |
| 25 | Missing data | 732 (0.1%) | 0 | -732 |

**Table S2:** Field size (km^2^) summary statistics per mapped reference area for all classes (1 – 4), definite burned classes (1 and 2), and unburned classes (4). All polygons were attributed with the following field classification: 1 = Active flame or burned area with corresponding VIIRS active fire point; 2 = Definite burned area but with no flame or active fire point; 3 = Ambiguous (a distinct darkening occurred on the field, but analyst is unsure if the field was burned then plowed or only plowed); 4 = Definitely unburned; 5 = Non-cropland or fields are too small that land cover conditions were difficult to determine on very high resolution (3 m) imagery.

|  | Reference Area | Number of Fields | Minimum | Maximum | Median | Mean | Standard Deviation |
| --- | --- | --- | --- | --- | --- | --- | --- |
| Class 1 - 4 | A | 3959 | 0.000* | 3.578 | 0.194 | 0.374 | 0.434 |
|  | B | 6167 | 0.001 | 8.657 | 0.380 | 0.577 | 0.591 |
|  | C | 8802 | 0.006 | 6.163 | 0.206 | 0.401 | 0.520 |
|  | D | 4905 | 0.006 | 3.916 | 0.286 | 0.480 | 0.492 |
|  | E | 5399 | 0.006 | 7.979 | 0.286 | 0.448 | 0.481 |
|  | F | 425 | 0.017 | 3.355 | 0.370 | 0.507 | 0.515 |
|  | G | 107 | 0.023 | 2.966 | 0.332 | 0.464 | 0.499 |
|  | Combined | 41883 | 0.000* | 8.657 | 0.218 | 0.412 | 0.492 |
|  | | | | | | | |
| Class 1 & 2 | A | 216 | 0.010 | 2.219 | 0.163 | 0.359 | 0.445 |
|  | B | 853 | 0.001 | 8.657 | 0.315 | 0.548 | 0.706 |
|  | C | 1228 | 0.009 | 5.396 | 0.214 | 0.393 | 0.494 |
|  | D | 714 | 0.022 | 3.231 | 0.279 | 0.491 | 0.522 |
|  | E | 425 | 0.017 | 3.355 | 0.370 | 0.507 | 0.515 |
|  | F | 107 | 0.023 | 2.966 | 0.332 | 0.464 | 0.499 |
|  | G | 4867 | 0.006 | 7.979 | 0.276 | 0.443 | 0.477 |
|  | Combined | 6897 | 0.001 | 8.657 | 0.182 | 0.374 | 0.497 |
|  | | | | | | | |
| Class 4 | A | 3526 | 0.000* | 3.578 | 0.195 | 0.377 | 0.435 |
|  | B | 4912 | 0.006 | 4.572 | 0.406 | 0.592 | 0.575 |
|  | C | 7457 | 0.006 | 6.163 | 0.206 | 0.405 | 0.525 |
|  | D | 4025 | 0.006 | 3.916 | 0.289 | 0.481 | 0.489 |
|  | E | 4867 | 0.006 | 7.979 | 0.276 | 0.443 | 0.477 |
|  | F | 2207 | 0.015 | 3.199 | 0.299 | 0.467 | 0.447 |
|  | G | 6709 | 0.004 | 5.145 | 0.117 | 0.265 | 0.378 |
|  | Combined | 33703 | 0.000* | 7.979 | 0.226 | 0.420 | 0.492 |

*The smallest field size digitized was 0.000031km^2^

**Table S3a** - 2016 monthly VIIRS filtered active fire counts. As a visual aid, grey entries represents monthly oblast active fire counts less than 50, and bold entries represent monthly active fire counts greater or equal to 50. The colored grid cells represent the majority crop type associated with the active fires. Green = maize, yellow = sunflower, orange = winter wheat, blue = soybean, brown = other cereals. The Oblasts have been sorted by highest total fire count.

|  | **Oblast** | **Jan** | **Feb** | **Mar** | **Apr** | **May** | **Jun** | **Jul** | **Aug** | **Sep** | **Oct** | **Nov** | **Dec** |
| --- | --- | --- | --- | --- | --- | --- | --- | --- | --- | --- | --- | --- | --- |
| **2016** | **Odes'ka** | 2 | 25 | 47 | 27 | 3 | 25 | **6758** | **1786** | **670** | **201** | 21 | 21 |
|  | **Donets'ka** | 5 | 39 | **76** | **141** | 36 | 32 | **1625** | **2612** | **546** | **119** | 37 | 3 |
|  | **Zaporiz'ka** | 1 | 25 | **63** | **66** | 6 | 7 | **1795** | **1658** | **519** | **99** | 4 | 0 |
|  | **Dnipropetrovs'ka** | 0 | 22 | **125** | **218** | 35 | 35 | **1739** | **993** | **616** | **462** | 33 | 1 |
|  | **Khersons'ka** | 1 | 6 | 42 | **52** | 0 | 18 | **1753** | **1065** | **293** | **156** | 17 | 5 |
|  | **Mykolayivs'ka** | 0 | 3 | **60** | 38 | 1 | 3 | **2007** | **535** | **438** | **119** | 5 | 0 |
|  | **Kharkivs'ka** | 0 | 11 | **54** | **364** | 20 | 16 | **405** | **1017** | **110** | **145** | 21 | 0 |
|  | **Kirovohrads'ka** | 1 | 7 | **100** | **166** | 25 | 12 | **1335** | **432** | **366** | **600** | 24 | 1 |
|  | **Vinnyts'ka** | 0 | 1 | **145** | **120** | 8 | 5 | **789** | **1784** | **499** | **354** | 4 | 0 |
|  | **Luhans'ka** | 0 | 3 | 32 | **151** | 1 | 0 | **286** | **900** | **221** | **65** | 16 | 0 |
|  | **Poltavs'ka** | 0 | 9 | **92** | **355** | 43 | 45 | **224** | **265** | **103** | **334** | 15 | 1 |
|  | **Khmel'nyts'ka** | 0 | 1 | **199** | **183** | 5 | 3 | **430** | **1382** | **311** | **89** | 0 | 0 |
|  | **Sums'ka** | 0 | 2 | **65** | **497** | 32 | 3 | 40 | **210** | **65** | **73** | 2 | 0 |
|  | **Cherkas'ka** | 0 | 0 | **94** | **159** | 7 | 4 | **216** | **510** | **278** | **370** | 4 | 0 |
|  | **Ternopil's'ka** | 0 | 0 | **92** | **56** | 2 | 0 | **207** | **992** | **224** | 20 | 0 | 0 |
|  | **Chernihivs'ka** | 0 | 5 | **101** | **260** | 21 | 1 | **112** | **309** | **59** | **52** | 2 | 0 |
|  | **Kyyivs'ka** | 1 | 1 | **114** | **221** | 7 | 2 | **166** | **356** | **217** | **184** | 6 | 0 |
|  | **L'vivs'ka** | 0 | 25 | **133** | **60** | 7 | 5 | 38 | **677** | **253** | 12 | 1 | 0 |
|  | **Rivnens'ka** | 1 | 2 | **70** | 31 | 4 | 9 | 44 | **581** | **640** | **79** | 1 | 0 |
|  | **Krym** | 2 | 14 | 44 | 32 | 0 | 8 | **123** | **148** | **110** | 24 | 8 | 0 |
|  | **Zhytomyrs'ka** | 0 | 2 | **124** | **101** | 5 | 0 | 45 | **322** | **150** | **65** | 0 | 0 |
|  | **Volyns'ka** | 0 | 8 | **79** | 17 | 3 | 8 | 14 | **314** | **101** | 25 | 0 | 0 |
|  | **Ivano-frankivs'ka** | 0 | 15 | 38 | 20 | 0 | 0 | **76** | **221** | 37 | 1 | 0 | 0 |
|  | **Chernivets'ka** | 1 | 1 | **71** | 34 | 0 | 0 | **94** | 31 | 35 | 5 | 0 | 1 |
|  | **Zakarpats'ka** | 0 | 0 | 4 | 3 | 0 | 1 | 3 | **60** | 17 | 1 | 0 | 0 |

**Table S3b** - 2017 monthly VIIRS filtered active fire counts. As a visual aid, grey entries represents monthly oblast active fire counts less than 50, and bold entries represent monthly active fire counts greater or equal to 50. The colored grid cells represent the majority crop type associated with the active fires. Green = maize, yellow = sunflower, orange = winter wheat, blue = soybean, brown = other cereals. The Oblasts have been sorted by highest total fire count.

|  | **Oblast** | **Jan** | **Feb** | **Mar** | **Apr** | **May** | **Jun** | **Jul** | **Aug** | **Sep** | **Oct** | **Nov** | **Dec** |
| --- | --- | --- | --- | --- | --- | --- | --- | --- | --- | --- | --- | --- | --- |
| **2017** | **Odes'ka** | 6 | 5 | **286** | **111** | 11 | 11 | **4705** | **2329** | **387** | **178** | 15 | 1 |
|  | **Donets'ka** | 2 | 3 | **443** | **301** | 33 | 25 | **1184** | **6300** | **697** | **133** | 5 | 2 |
|  | **Zaporiz'ka** | 0 | 3 | **183** | **81** | 21 | 47 | **2223** | **3824** | **668** | **63** | 10 | 0 |
|  | **Dnipropetrovs'ka** | 2 | 2 | **816** | **351** | **72** | 39 | **2111** | **2188** | **431** | **228** | 16 | 6 |
|  | **Khersons'ka** | 0 | 21 | **292** | **102** | 18 | **66** | **2795** | **2031** | **451** | **342** | 39 | 1 |
|  | **Mykolayivs'ka** | 0 | 0 | **132** | **74** | 11 | 14 | **1919** | **833** | **264** | **61** | 0 | 0 |
|  | **Kharkivs'ka** | 1 | 0 | **641** | **859** | **141** | 29 | **418** | **1706** | **302** | **178** | 6 | 4 |
|  | **Kirovohrads'ka** | 4 | 6 | **770** | **441** | **108** | **51** | **707** | **408** | **349** | **265** | 2 | 2 |
|  | **Vinnyts'ka** | 6 | 0 | **268** | **387** | **50** | 5 | **388** | **963** | **100** | **126** | 9 | 0 |
|  | **Luhans'ka** | 0 | 0 | **353** | **486** | **103** | 11 | **694** | **2018** | **370** | **93** | 1 | 0 |
|  | **Poltavs'ka** | 4 | 1 | **1391** | **1221** | **191** | **67** | **186** | **189** | **139** | **180** | 5 | 1 |
|  | **Khmel'nyts'ka** | 0 | 0 | **123** | **289** | 24 | 2 | 43 | **917** | **104** | 12 | 0 | 0 |
|  | **Sums'ka** | 0 | 0 | **772** | **869** | **121** | 15 | 16 | **276** | 48 | 31 | 1 | 0 |
|  | **Cherkas'ka** | 0 | 1 | **524** | **290** | **51** | 11 | **75** | **147** | **67** | **94** | 4 | 0 |
|  | **Ternopil's'ka** | 0 | 3 | **52** | **100** | 2 | 3 | 15 | **629** | **103** | 42 | 0 | 0 |
|  | **Chernihivs'ka** | 0 | 0 | **472** | **387** | 48 | 12 | 33 | **273** | **50** | 22 | 0 | 0 |
|  | **Kyyivs'ka** | 0 | 1 | **329** | **269** | **58** | 20 | 48 | **130** | 48 | 26 | 0 | 0 |
|  | **L'vivs'ka** | 0 | **78** | **102** | **95** | 1 | 4 | 6 | **365** | 20 | 12 | 0 | 0 |
|  | **Rivnens'ka** | 1 | 0 | 48 | **75** | 13 | 0 | 7 | **115** | 16 | 2 | 0 | 0 |
|  | **Krym** | 0 | 34 | **193** | 38 | 5 | 33 | **170** | **353** | **179** | **51** | 0 | 2 |
|  | **Zhytomyrs'ka** | 0 | 1 | **94** | **224** | 28 | 5 | 5 | **120** | 24 | 0 | 1 | 0 |
|  | **Volyns'ka** | 0 | 6 | **56** | **91** | 3 | 6 | 1 | **93** | 12 | 5 | 0 | 0 |
|  | **Ivano-frankivs'ka** | 0 | 46 | **56** | 34 | 0 | 0 | 7 | **109** | 8 | 1 | 0 | 0 |
|  | **Chernivets'ka** | 1 | 4 | 31 | 12 | 1 | 0 | 26 | **144** | 3 | 14 | 0 | 0 |
|  | **Zakarpats'ka** | 0 | 0 | 22 | 25 | 2 | 1 | 2 | 10 | 4 | 1 | 0 | 0 |

**Table S4** – 2016 and 2017 combined total active fire, 2016 and 2017 combined burned area and percent cropland in 2017 summarized per Oblast. Oblasts are ranked by the total number of cropland VIIRS active fires in 2016 and 2017. Total 2016 and 2017 burned area (km^2^) estimates are based on the average high (α_H_) and low (α_L_) conversion factors. Spring conversion factors were applied to active fire counts between January and June, while summer conversion factors were applied to active fire counts between July and December.

| Oblast | Rank | 2016 and 2017 Total Active Fire Count | 2016 and 2017 Total Minimum  Burned Area (km^2^) | 2016 and 2017 Total Maximum  Burned Area (km^2^) | 2017 Cropland (%) |
| --- | --- | --- | --- | --- | --- |
| Odes'ka | 1 | 17631 | 8658 | 10072 | 61 |
| Donets'ka | 2 | 14399 | 7137 | 8467 | 51 |
| Zaporiz'ka | 3 | 11366 | 5595 | 6544 | 72 |
| Dnipropetrovs'ka | 4 | 10541 | 5311 | 6512 | 64 |
| Khersons'ka | 5 | 9566 | 4728 | 5576 | 65 |
| Mykolayivs'ka | 6 | 6517 | 3213 | 3769 | 69 |
| Kharkivs'ka | 7 | 6448 | 3354 | 4369 | 58 |
| Kirovohrads'ka | 8 | 6182 | 3181 | 4062 | 68 |
| Vinnyts'ka | 9 | 6011 | 3030 | 3719 | 57 |
| Luhans'ka | 10 | 5804 | 2943 | 3655 | 43 |
| Poltavs'ka | 11 | 5061 | 2801 | 4048 | 59 |
| Khmel'nyts'ka | 12 | 4117 | 2090 | 2600 | 50 |
| Sums'ka | 13 | 3138 | 1762 | 2601 | 48 |
| Cherkas'ka | 14 | 2906 | 1529 | 2032 | 57 |
| Ternopil's'ka | 15 | 2542 | 1271 | 1534 | 46 |
| Chernihivs'ka | 16 | 2219 | 1210 | 1707 | 36 |
| Kyyivs'ka | 17 | 2204 | 1175 | 1597 | 39 |
| L'vivs'ka | 18 | 1894 | 974 | 1242 | 29 |
| Rivnens'ka | 19 | 1739 | 873 | 1064 | 23 |
| Krym | 20 | 1571 | 806 | 1023 | 40 |
| Zhytomyrs'ka | 21 | 1316 | 699 | 944 | 21 |
| Volyns'ka | 22 | 842 | 438 | 570 | 24 |
| Ivano-frankivs'ka | 23 | 669 | 347 | 449 | 17 |
| Chernivets'ka | 24 | 509 | 264 | 340 | 21 |

**SI References**

1. Drusch, M. *et al.* Sentinel-2: ESA’s Optical High-Resolution Mission for GMES Operational Services. *Remote Sens. Environ.* **120**, 25–36 (2012).

2. Roy, D. P. *et al.* Landsat-8: Science and product vision for terrestrial global change research. *Remote Sens. Environ.* **145**, 154–172 (2014).

3. Torres, R. *et al.* GMES Sentinel-1 mission. *Remote Sens. Environ.* **120**, 9–24 (2012).

4. Kussul, N. *et al.* Parcel-Based Crop Classification in Ukraine Using Landsat-8 Data and Sentinel-1A Data. *IEEE J. Sel. Top. Appl. Earth Obs. Remote Sens.* **9**, 2500–2508 (2016).

5. Kussul, N., Lavreniuk, M., Skakun, S. & Shelestov, A. Deep Learning Classification of Land Cover and Crop Types Using Remote Sensing Data. *IEEE Geosci. Remote Sens. Lett.* **14**, 778–782 (2017).

6. Shelestov, A., Lavreniuk, M., Kussul, N., Novikov, A. & Skakun, S. Exploring Google earth engine platform for big data processing: Classification of multi-temporal satellite imagery for crop mapping. *Front. Earth Sci.* **5**, 1–10 (2017).

7. Shelestov, A. *et al.* Cloud Approach to Automated Crop Classification Using Sentinel-1 Imagery. *IEEE Trans. Big Data* 1–1 (2019) doi:10.1109/tbdata.2019.2940237.

8. Skakun, S., Kussul, N., Shelestov, A. Y., Lavreniuk, M. & Kussul, O. Efficiency Assessment of Multitemporal C-Band Radarsat-2 Intensity and Landsat-8 Surface Reflectance Satellite Imagery for Crop Classification in Ukraine. *IEEE J. Sel. Top. Appl. Earth Obs. Remote Sens.* **9**, 3712–3719 (2016).

9. Waldner, F. *et al.* Towards a set of agrosystem-specific cropland mapping methods to address the global cropland diversity. *Int. J. Remote Sens.* **37**, 3196–3231 (2016).

10. Waldner, F. *et al.* Roadside collection of training data for cropland mapping is viable when environmental and management gradients are surveyed. *Int. J. Appl. Earth Obs. Geoinf.* **80**, 82–93 (2019).

11. Kingma, D. P. & Ba, J. L. Adam: A method for stochastic optimization. *3rd Int. Conf. Learn. Represent. ICLR 2015 - Conf. Track Proc.* 1–15 (2015).

12. Schroeder, W., Oliva, P., Giglio, L. & Csiszar, I. A. The New VIIRS 375m active fire detection data product: Algorithm description and initial assessment. *Remote Sens. Environ.* **143**, 85–96 (2014).

13. Giglio, L. MODIS Collection 6 Active Fire Product User’s Guide. (2015). <http://modis-fire.umd.edu/files/MODIS_C6_Fire_User_Guide_A.pdf>. Last accessed 21^st^ August 2020.

14. Giglio, L., van der Werf, G. R., Randerson, J. T., Collatz, G. J. & Kasibhatla, P. Global estimation of burned area using MODIS active fire observations. *Atmos. Chem. Phys.* **6**, 957–974 (2006).
